# Evaluation of the effectiveness of Washington State’s digital COVID-19 exposure notification system over one pandemic year

**DOI:** 10.3389/fpubh.2024.1408178

**Published:** 2024-08-14

**Authors:** Adam S. Elder, Cory J. Arrouzet, Ljubomir Miljacic, Bryant T. Karras, Amanda Higgins, Laura M. West, Daniel Lorigan, Debra Revere, Nayak Polissar, Courtney D. Segal, William B. Lober, Janet G. Baseman

**Affiliations:** ^1^Department of Epidemiology, School of Public Health, University of Washington, Seattle, WA, United States; ^2^The Mountain-Whisper-Light: Statistics & Data Science, Seattle, WA, United States; ^3^Washington State Department, Tumwater, WA, United States; ^4^Department of Biobehavioral Nursing and Health Informatics, School of Nursing, University of Washington, Seattle, WA, United States; ^5^Department of Health Systems and Population Health, School of Public Health, University of Washington, Seattle, WA, United States

**Keywords:** digital exposure notifications, data privacy, non-pharmaceutical interventions, COVID-19, outbreak investigation and response

## Abstract

**Introduction:**

Digital exposure notifications are a novel public health intervention used during the COVID-19 pandemic to alert users of possible COVID-19 exposure. We seek to quantify the effectiveness of Washington State’s digital exposure notification system, WA Notify, as measured by the number of COVID-19 cases averted during a 1-year period.

**Methods:**

While maintaining individuals’ privacy, WA Notify collected data that could be used to evaluate the system’s effectiveness. This article uses these and other data and builds on a previous model to estimate the number of cases averted by WA Notify. Novel estimates of some model parameters are possible because of improvements in the quality and breadth of data reported by WA Notify.

**Results:**

We estimate that WA Notify averted 64,000 (sensitivity analysis: 35,000–92,000) COVID-19 cases in Washington State during the study period from 1 March 2021 to 28 February 2022. During this period, there were an estimated 1,089,000 exposure notifications generated and 155,000 cases reported to WA Notify. During the last 78 days of the study period, the median estimated number of daily active users was 1,740,000.

**Discussion:**

We believe WA Notify reduced the impact of the COVID-19 pandemic in Washington State and that similar systems could reduce the impact of future communicable disease outbreaks.

## 1 Introduction

Early in the SARS-CoV-2 pandemic, challenges to traditional public health case investigation/contact tracing (CI/CT) were widely acknowledged, including limited surge contact tracing capacity, inability to identify contacts unknown to individuals who tested positive, and the unwillingness of the public to respond to contact tracing phone or text outreach (1). In early 2020, new tools using Bluetooth technology were developed using the Google–Apple Exposure Notification (GAEN) system (2), which followed Privacy-sensitive protocols And mechanisms for mobile Contact Tracing (PACT) guidelines (3). These tools became available to public health and governmental organizations to help slow the spread of COVID-19. In Washington State (WA), an implementation of the express version of the GAEN system, named WA Notify, was created. Because WA Notify was embedded in the operating system of smartphones, it was easy for users to enable. After a pilot study, WA Notify was both endorsed and implemented statewide by the by the Washington State Department of Health (WA DOH) to supplement the state’s CI/CT program (4). Similar digital exposure notification systems built using GAEN were introduced in 28 states inside of the United States and at least 38 countries around the world, including the United Kingdom (5).

Smartphone-based exposure notification (EN) systems and traditional CI/CT programs both aim to rapidly inform close contacts of individuals who test positive for COVID-19 that they may have been exposed to the virus. These two approaches were often directly compared, with EN systems referred to as “digital contact tracing systems (6–8).” However, these interventions utilize distinct mechanisms for informing individuals about a potential exposure. Traditional contact tracing relies on existing public health resources to identify and notify contacts of their possible exposure to an index case who has tested positive. A digital EN tool relies instead on its users to self-attest a positive test result using their smartphone and allows for the notification of possible exposure to other users who have been in close proximity to the individual who has tested positive during the infectious period. Like other digital EN tools, WA Notify was deployed before knowing its effectiveness, although early analyses indicated that digital EN systems may mitigate the spread of COVID-19 (9, 10).

While the privacy preservation and data protections in EN systems present challenges to evaluating the EN systems’ effectiveness, this article builds on prior studies that characterized metrics for investigating the effectiveness of such EN systems (10, 11). This study presents an update to earlier analyses of WA Notify (10). While both analyses estimate the number of cases averted by WA Notify, this study considers a longer 1-year study period from 1 March 2021 to 28 February 2022 and uses an updated estimation strategy.

This analysis quantifies the effectiveness of WA Notify as measured by the number of COVID-19 cases averted during a 1-year period. In the next section, we provide a description of the WA Notify system, and the data and model used to estimate the number of cases averted by the system. In the third section, we provide our estimate of cases averted by WA Notify, and in the fourth section, we discuss the limitations and implications of the results found in our analysis. In the final section we provide concluding remarks. The Supplementary material provides additional details about the methods used and sensitivity analyses conducted to evaluate the robustness of our model.

## 2 Materials and methods

### 2.1 The WA Notify system

Washington State residents were encouraged to enroll in WA Notify by enabling the system on their Android or iOS smartphones. Multiple communication strategies were leveraged to encourage adoption, including notifications that appeared on iOS and Android smartphones and press releases from the governor’s office and state/local departments of health (12). While any individual living in Washington State (even including individuals visiting for short periods) would have been able to activate the system on their device, we believe the number of cases averted by the system for interactions happening outside of the state is negligible and this possibility will be ignored in this analysis.

The WA Notify system was a collection of computer servers providing the infrastructure to inform users of potential exposures to COVID-19. Users learned about their exposure via a notification that appeared on their phone—much like other smartphone notifications. The WA Notify system heavily relied on the use of modern cryptographic systems to maintain the privacy of individuals and protect the accuracy of usage metrics. These metrics were reported by the system to public health authorities and researchers. Both the “codes” and “keys” discussed later in this article were privacy-preserving alphanumeric strings that were shared between users’ devices and WA Notify servers to record the close proximity of users’ phones or to report a user’s positive test result.

### 2.2 The Exposure Notification Code Verification system

The Exposure Notification Code Verification (ENCV) system was a component of WA Notify that facilitated the four-step process required for a phone to display an exposure notification (EN) to the user. For an EN to be generated on a user’s phone, it was necessary that other users carried out certain actions that are outlined below, though they could choose not to take these actions. The four required steps for an EN to be generated were as follows:

1. Two phones that had enrolled in WA Notify (or another exposure notification express system) were close enough to one another to exchange Bluetooth keys. For example, the CDC originally defined a close contact as a person who was less than 6 feet away from an infected individual for at least 15 min.
2. Once a user received a positive COVID-19 diagnosis, they chose to receive a code—referred to as a verification code—from the ENCV system. The user then also chose to confirm their diagnosis with the ENCV system using a process referred to as code verification (CV).
3. After completing code verification, the user chose to upload the Bluetooth keys that had been broadcasted by their smartphone over the last 14 days to the National Key Server (NKS). The process of uploading keys to the NKS is referred to here as “publishing keys.”
4. Multiple times a day, phones enrolled in Exposure Notification express systems automatically downloaded newly published keys from the NKS. After downloading the published keys, the phone searched for a match between the published keys and those collected by the phone from prior Bluetooth key exchanges over the previous 10 days. Prior to January 2022, keys from the last 14 days were compared. Based on the number of matched keys and the proximity and length of the associated interactions, an EN may have been generated to be shown to the user. Note that matching was carried out on phones, so the ENCV system could not determine which users had been exposed.

**Figure 1:**
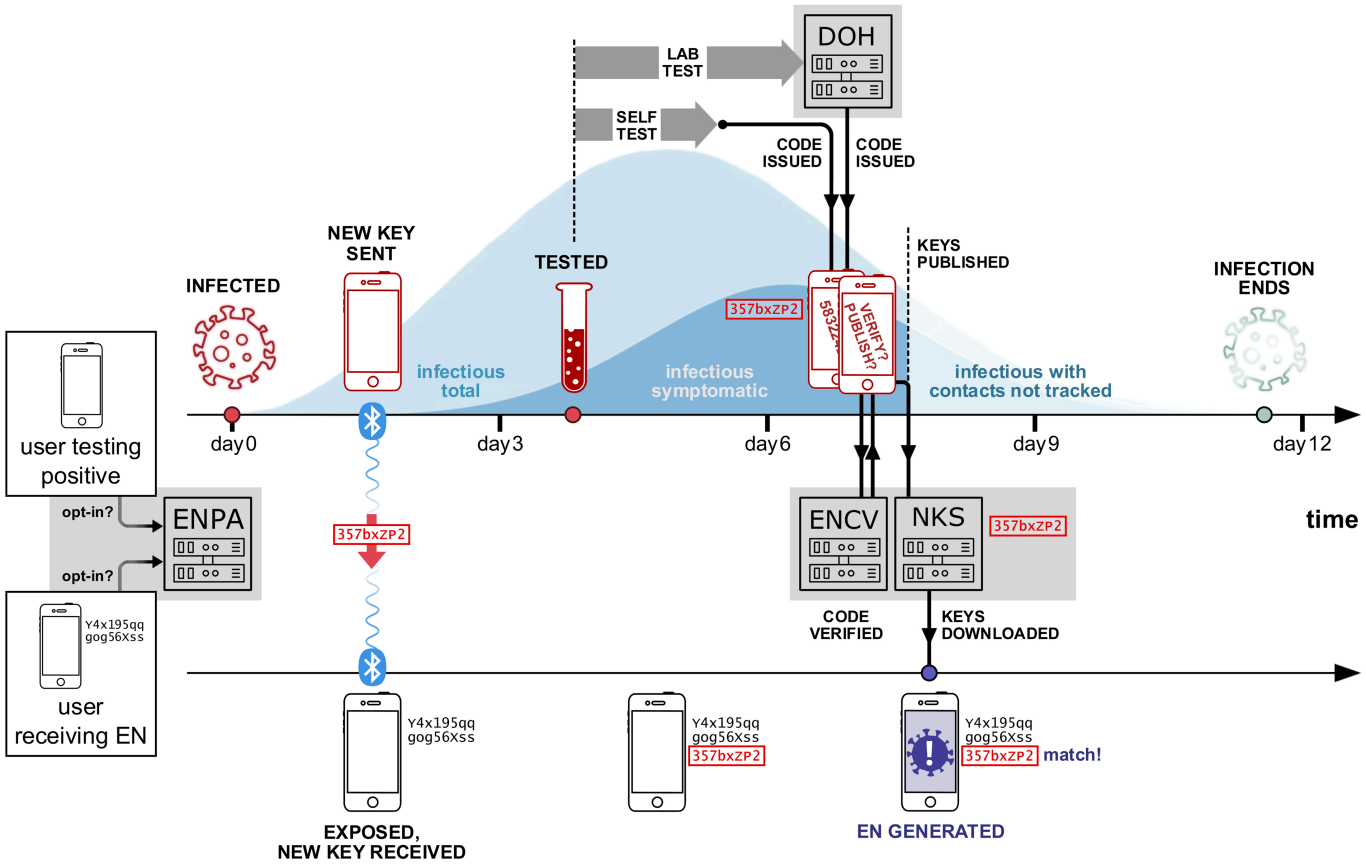
Simplified example of the WA Notify system interacting with two phones (and two participants). Each opted-in user sends a daily summary (including interactions with other phones enrolled in WA Notify) to the ENPA system. The first phone (shown in the upper timeline) belongs to a user who was infected on day 0. The second phone (lower timeline) belongs to a user who was in close proximity to the first user at some time during days 0 to 3. During the period of close proximity, a Bluetooth key is created on the infected user’s phone and sent to the exposed user’s phone. The key is stored on both phones for future reference. On day 4, the first user tests for COVID-19. Next, they receive their positive test results and subsequently are issued a code on day 7, either by DOH or via self-attestation. After being issued a code, the user consents to verify their code with the exposure notification code verification (ENCV) server and then publishes their keys on the national key server (NKS). Multiple times each day, published keys are downloaded onto all WA Notify phones, including the second user’s phone. After downloading the newly published keys, the second user’s phone finds a match between the newly published keys and a key already stored on the phone from the previous interaction with the first user’s phone. This leads to an EN being generated on the second user’s phone.

Figure 1 provides an example of how this process could unfold for two WA Notify users.

### 2.3 The Exposure Notification Privacy-Preserving Analytics system

Once enrolled in WA Notify, users could opt-in to provide additional anonymized information to the Exposure Notification Privacy-preserving Analytics (ENPA) system. This included the number of ENs generated and displayed on the opted-in user’s phone. The ENPA system employed multiple layers of security to maintain individuals’ data privacy (13). More detailed technical descriptions of both the ENCV and ENPA systems can be found in the Supplementary material and on the Apple/Google “Privacy-Preserving Contact Tracing” website (14).

### 2.4 Data sources

This analysis utilizes four primary data sources: ENCV server data, ENPA system data, WA DOH COVID-19 case counts and sequencing data, and the WA Notify User Survey data, each of which is described in further detail below.

Among other metrics, ENCV server data provided exact counts of the number of code verifications (CVs) and keys published across all WA Notify user cell phones. The number of code verifications recorded by the ENCV server is the number of WA Notify users who had successfully confirmed their positive COVID-19 test results with WA Notify. Because key matching and risk assessment were carried out on users’ phones, the number of generated ENs cannot be estimated using ENCV server data alone.

The second data source, the ENPA system, provides additional aggregate counts beyond metrics reported by the ENCV, though all counts reported by the ENPA are limited to individuals who had opted into the system. This includes approximate counts of the number of ENs generated on users’ phones, CVs that followed an EN being generated on the phone in the past 10 days, and CVs that did not follow an EN.

The third data source, the WA DOH COVID-19 case count and sequencing counts, is accessible from the WA DOH website (15), which provides the 7-day moving average of COVID-19 case counts in Washington State. In addition, each week, a subset of positive samples is sequenced to determine the COVID-19 variant of the sample. These variant counts are reported on the website and can be used to estimate the percentage of cases attributable to each COVID-19 variant.

Finally, the WA Notify User Survey was an online survey accessible by individuals who opened an EN on their phones. Users were taken to a DOH webpage providing public health guidance for recent exposures, as well as a link to the WA Notify User Survey. The baseline survey captured feedback on users’ experiences with WA Notify and planned engagement in protective behaviors. After this survey, respondents were asked whether they were willing to receive a follow-up survey. Those who agreed and provided their email address received an email invitation to complete a follow-up survey 2 weeks after completing the baseline survey. The follow-up survey included questions about the protective behaviors taken by participants after the EN appeared on their phones (16).

### 2.5 Model structure

**Figure 2:**
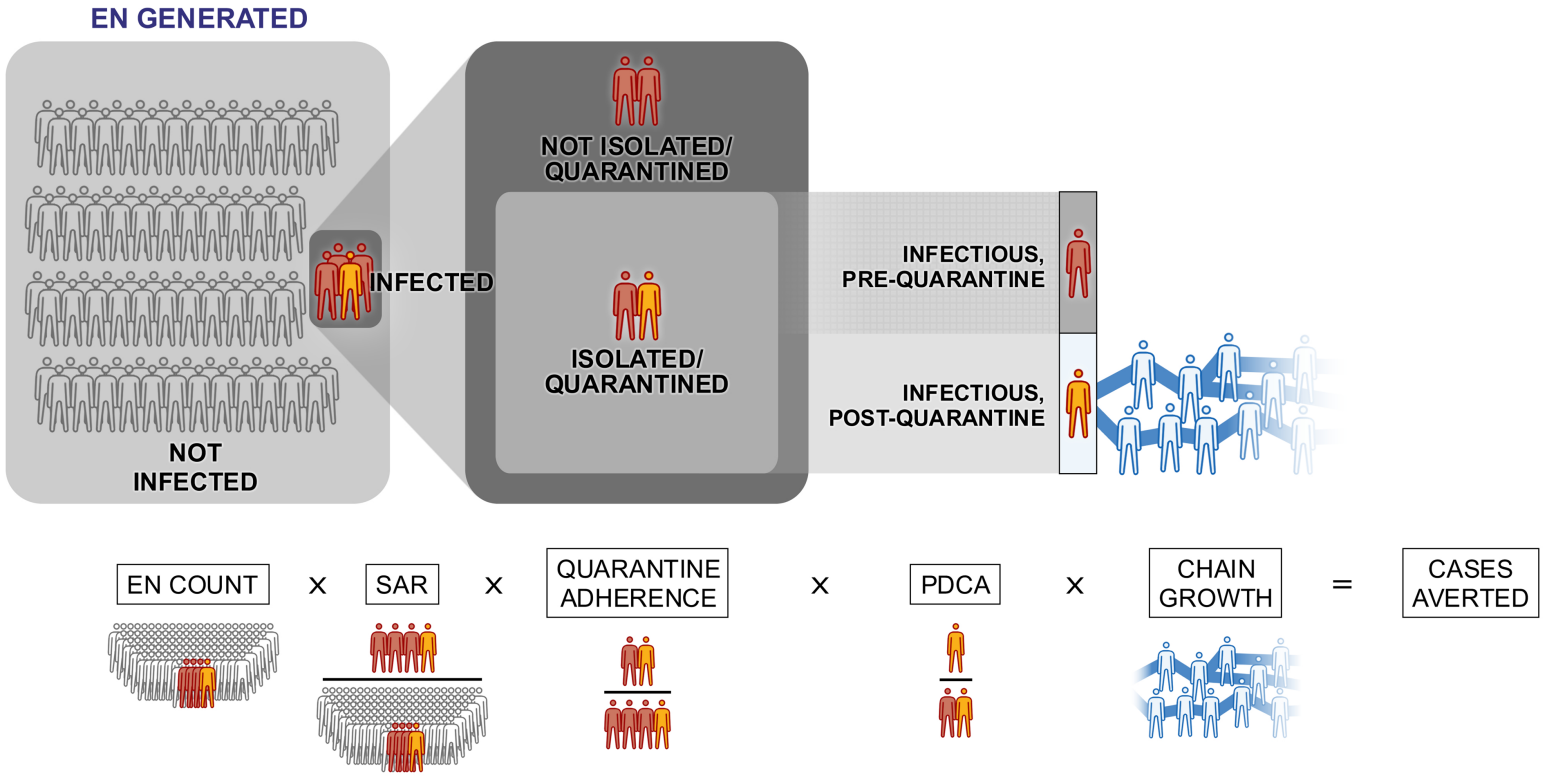
Visual representation of the five parameters used to calculate the daily number of cases averted. EN stands for exposure notification, SAR stands for (App-Based) Secondary Attack Rate, and PDCA stands for Proportion of direct cases averted.

To evaluate WA Notify’s effectiveness, the number of cases averted by the system in Washington State is estimated. We define the number of cases averted as the number of *additional* cases of COVID-19 that would have occurred in Washington State had the WA Notify system not been implemented, but all other interventions (including traditional CI/CT) had been carried out as they were. While many states did not implement digital exposure notification systems, suggesting comparisons of case counts between these states and Washington could be used to estimate cases averted by the system, such comparisons would do a poor job distinguishing the effects of WA Notify from other factors that effected each state’s case counts such as population density, political climate, and level of vaccine adoption. This between-state comparison would have limited causal interpretability because these and other factors impacting case counts also likely impact each state’s decisions regarding the adoption of a digital exposure notification system. Instead, the estimation strategy used in this study is an adaptation of a modeling approach proposed by Wymant et al. (9). The model used here allows for the estimation of cases averted and implicitly proposes an individual-level process by which WA Notify would avert a COVID-19 case. The model also proposes a set of assumptions, under which it is possible to estimate the effect of this individual-level process at the population level in Washington State. Figure 2 illustrates the five population-level parameters of the model and how they connect with the five-step individual-level process outlined below. In addition to the assumptions described below, each model parameter estimator makes certain transportability and independence assumptions, which are summarized in Table 1 and are described in more detail in the Supplementary material.

**Table 1:** Five model parameters, their definitions, associated assumptions, data sources used, and the corresponding average estimates during three different periods of the study (the mean taken across days in the time period).

Model input	Number of exposure notifications (EN)	App-based secondary attack rate (SAR)	Quarantine adherence (QA)	Proportion of direct cases averted (PDCA)	Chain size (CS)
Description	Exposure notifications appearing on individuals’ phones	The ratio of the number of ENs observed by infected individuals to ENs observed overall	The proportion of individuals quarantining after observing an EN	The proportion of potential infections prevented by quarantining after observing an EN	The average size of the infection chain starts on the given day and ends at the end of the study period.
Cumulative product meaning	Number of ENs	Number of notified individuals who are infected	Number of notified and infected individuals who are quarantined	Number of direct cases averted	Number of cases averted
Assumptions and (Hypothesized bias); estimator over(estimates) or under(estimates) parameter		Only individuals who report positive status to WA Notify are infected (under)	Individuals quarantine as a result of EN (over) Infections are prevented only by quarantine (under)	Individuals quarantine immediately after observing an EN (over)	Susceptible individuals are part of at most one infection chain at a time (over). See Supplementary material for details.
Transferability assumption (the population of the estimator is similar to the population of the parameter)	ENPA^a^ population is similar to the WA Notify population	ENPA^a^ population is similar to the WA Notify population	The survey population (those who responded to the survey after EN) is similar to infected users who observed an EN	ENPA^a^ population is similar to the WA Notify population	WA Notify population is similar to the WA state population in chain size.
Independence assumption	Assumes each random variable defining the corresponding model parameter is mutually independent.				
Data sources	ENCV and ENPA^a^	ENCV and ENPA^a^	WA Notify User Survey	ENCV^a^ and ENPA^a^	WA DOH^a^ daily case counts and sequencing data
Alpha period 1 March–30 June 2021 96,000 cases	28,000	0.037	0.56 (0.3, 0.8)^b^	0.40	11^c^
Delta period 1 July–30 Nov 2021 339,000 cases	162,000	0.016		0.32	27^c^
Omicron period 1 Dec 2021–28 Feb 2022 670,000 cases	899,000	0.022		0.53	7^c^

a DOH stands for Department of Health, ENCV stands for Exposure Notification Code Verification, and ENPA stands for Exposure Notification Privacy Analytics.

b See the “Sensitivity to Quarantine Adherence” section of the Supplementary material for more details.

c Averages for chain size are weighted by the number of cases attributed to the given variant each day rather than giving each day an equal weight (like each other reported parameter average).

For each period, the number of total cases reported to the DOH during the period is listed in parentheses. The set of assumptions listed is necessary to guarantee that the value of the estimator will approach that of the parameter as the sample size grows in all settings. For some assumptions, an informed hypothesis can be made to the direction of the bias. In such cases, the direction of the bias is also indicated. The cumulative product meaning is the interpretation of the product of all parameters to the left of and including the given column. As an example, the cumulative product meaning of quarantine adherence is the interpretation of the product of EN, SAR, and QA for a given day. The assumption row lists the non-transferability and independence assumptions made when estimating the given parameter. For more discussion of these assumptions and the resulting biases that are possible, see the Supplementary material. The transferability assumption row describes the population used to estimate the parameter and the population of interest (the population used to define the parameter).

#### 2.5.1 Step one: an EN is observed on an individual’s phone

First, an EN must be displayed on an individual’s device. The corresponding model parameter is the number of ENs observed across all WA Notify devices. This number is estimated using the ENCV and ENPA data.

#### 2.5.2 Step two: the EN is observed by an infected individual

The second step in the individual-level process requires that the EN is observed by an infected individual. The second model parameter is the App-Based Secondary Attack Rate (SAR) which translates the total number of ENs observed to the number of ENs observed by infected users. The SAR is the ratio of ENs observed on infected users’ phones to ENs generated across all WA Notify users’ phones. The SAR is estimated as the ratio of the number of users who are shown an EN prior to reporting a positive COVID-19 test to WA Notify to the number of users shown an EN. The numerator of this ratio excludes individuals who are infected but do not report their status to WA Notify, either because they do not test or because they test positive but do not report the test to WA Notify. Excluding these COVID-19 cases biases the cases averted estimator downward.

#### 2.5.3 Step three: the EN prompts the individual to quarantine

Next, the infected individual responds to the EN by quarantining. The third model parameter, quarantine adherence (QA), is used alongside EN counts and SAR to estimate the number of individuals who satisfy the first three requirements of the individual-level process (observes EN, is infected, and quarantines). The QA parameter is the ratio of users who are both infected and notified and who quarantine in response to the EN to all users who are both infected and notified. This ratio is estimated from the WA Notify user survey. Survey respondents are counted as quarantine adherents if they report staying home for the recommended period of time or until they receive results from a COVID-19 test. The estimate of QA is the proportion of respondents (out of all respondents) who are categorized as quarantine adherents.

There are many possible reasons why the estimate for quarantine adherence could be larger than the corresponding parameter. Four of these include the following: (1) Individuals may have reported engaging in protective behaviors more frequently than they actually did; (2) individuals who quarantined may have been more likely to respond to the survey; (3) partial quarantine (for example staying home, but potentially interacting with housemates) was counted as full quarantine by our model; and (4) the model assumes that users who were both infected and notified and went on to quarantine did so because they observed an EN even though some individuals would quarantine for other reasons, such as becoming symptomatic.

Conversely, the true value of quarantine adherence could be larger than the estimate because of other sources of error. Individuals who are infectious and respond to the survey could be more likely to quarantine than the population of survey respondents observing an EN who may or may not be infectious. As an example, infected individuals, on average, have engaged in riskier behavior and may compensate by quarantining more frequently. Another reason why our estimate may be an underestimate is that individuals, after observing an EN, may not quarantine (and thus be counted as not adherent) but could take other protective actions not listed on the survey such as masking or canceling higher risk plans like going to a concert. Such behaviors would avert some infections but are not included in the definition of quarantine adherence used here.

Thus, while the proportion of those reporting to be quarantine adherent on the survey can be estimated accurately, there are high levels of uncertainty about what the true value of the model parameter is. This uncertainty arises from differences between the measurement (reported protective behaviors) and value of interest (reduction in interactions that could result in transmission). The model implicitly assumes that reported protective behaviors and reduction in interactions of infected individuals are the same.

Due to the significant uncertainty of the bias of the estimate, and the possible differences between reported QA and the actual reduction in interactions, the sensitivity analysis considers a wide range of values for QA. To select upper and lower bounds for QA, a variety of studies were considered (9, 17–25). After consideration of the survey data and these studies, a lower bound of 30% and an upper bound of 80% were selected.

#### 2.5.4 Step four: the EN is observed before the potential direct infection

For individuals who do quarantine, direct transmissions occurring before the EN are not averted, whereas those occurring after the EN are averted. The fourth model parameter accounts for the timeliness of the EN and is referred to as the proportion of direct cases averted (PDCA). The PDCA can be thought of as the fraction of all possible exposures that occur after the EN is observed. The PDCA is calculated as the probability that the time from infection to EN is less than the time from infection to subsequent infection (generation time). While the distribution of time from infection to EN is estimated using WA Notify users, estimates for generation time are taken from studies on other populations (26, 27). The model assumes that infected users who observe an EN and quarantine do so immediately which biases the estimator upward.

#### 2.5.5 Step five: subsequent infections

For each direct case that is averted because of behavior change caused by WA Notify, all subsequent infections that would have resulted from the case that was averted will also be averted. Calculating the exact size of such a chain of infections is difficult. However, the average size of a chain of infections, referred to here as chain size, can be estimated using case count and sequencing data provided by the state.

The chain size is the average number of future infections caused in a single chain of infections starting on the day of infection and ending the day the study ends. Accurate estimation of this parameter does not require that all cases are reported so long as the proportion of cases reported is constant during the study period. If susceptible individuals are part of multiple infection chains, then using chain size in the cases averted model to estimate future cases averted by directly averting a single case could bias the cases averted estimator upward. As an example, if an individual would be infected by either of two exposures and only one exposure is prevented by WA Notify, the case would not be averted.

While not explicitly included in the model, many environmental factors are accounted for because their effects on the number of cases averted are captured by the model parameters themselves. As an example, vaccine coverage can change the number of cases averted by WA Notify by increasing immunity. Changes resulting from higher vaccine coverage are accounted for by the model in the calculation of the SAR and chain size because the effects of vaccination are present in the ENPA population used to estimate the SAR and in incidence data from the WA DOH. In addition, for the estimation of the model parameters, the population defining the parameters is either used directly or a substantial subset of this population is used. This suggests a more representative sample and more accurate accounting of these environmental factors than if data came from an outside population.

For each day and variant, the number of cases averted is modeled as the product of the five model parameters for that day and variant. Each variant is considered separately in these calculations. Thus, the number of future cases the model attributes to an individual will be naturally limited to the period in which the given variant is present. For this study, direct cases averted prior to December 2021 will be modeled as averting few, if any, cases after December 2021 since a new variant (Omicron) crowded out other variants starting in December 2021.

Due to the strong, untestable assumptions made to translate direct cases averted to total cases averted using chain size, the number of direct cases averted is also reported. The direct cases averted include only transmissions that would have occurred between a WA Notify user and someone else (had the user not changed their behavior). Thus, no assumptions about how many future cases would result from this one transmission are made when calculating direct cases averted. All other assumptions made for estimating total cases averted are still made when estimating direct cases averted.

### 2.6 Sensitivity analysis

We conducted multiple sensitivity analyses to study how uncertainty in various parameter estimates would impact our estimate of cases averted. Considering all analyses, the variability in the estimate of cases averted is primarily driven by uncertainties in the quarantine adherence parameter. For simplicity, the primary sensitivity analysis presented in the main text estimates the number of cases averted for three different quarantine adherence levels. The uncertainty in our cases averted estimate coming from uncertainty in other parameters is considered in secondary sensitivity analyses presented in the Supplementary material. For the primary sensitivity analysis, the estimate of quarantine adherence is 56% with a lower bound of 30% and an upper bound of 80%. Each bound represents our belief of a reasonable limit for quarantine adherence based on the reviewed literature and responses to the WA Notify Survey.

### 2.7 Ethics

The University of Washington Institutional Review Board determined that this project was a public health quality improvement/surveillance project and therefore was deemed not human subjects research.

## 3 Results

**Figure 3:**
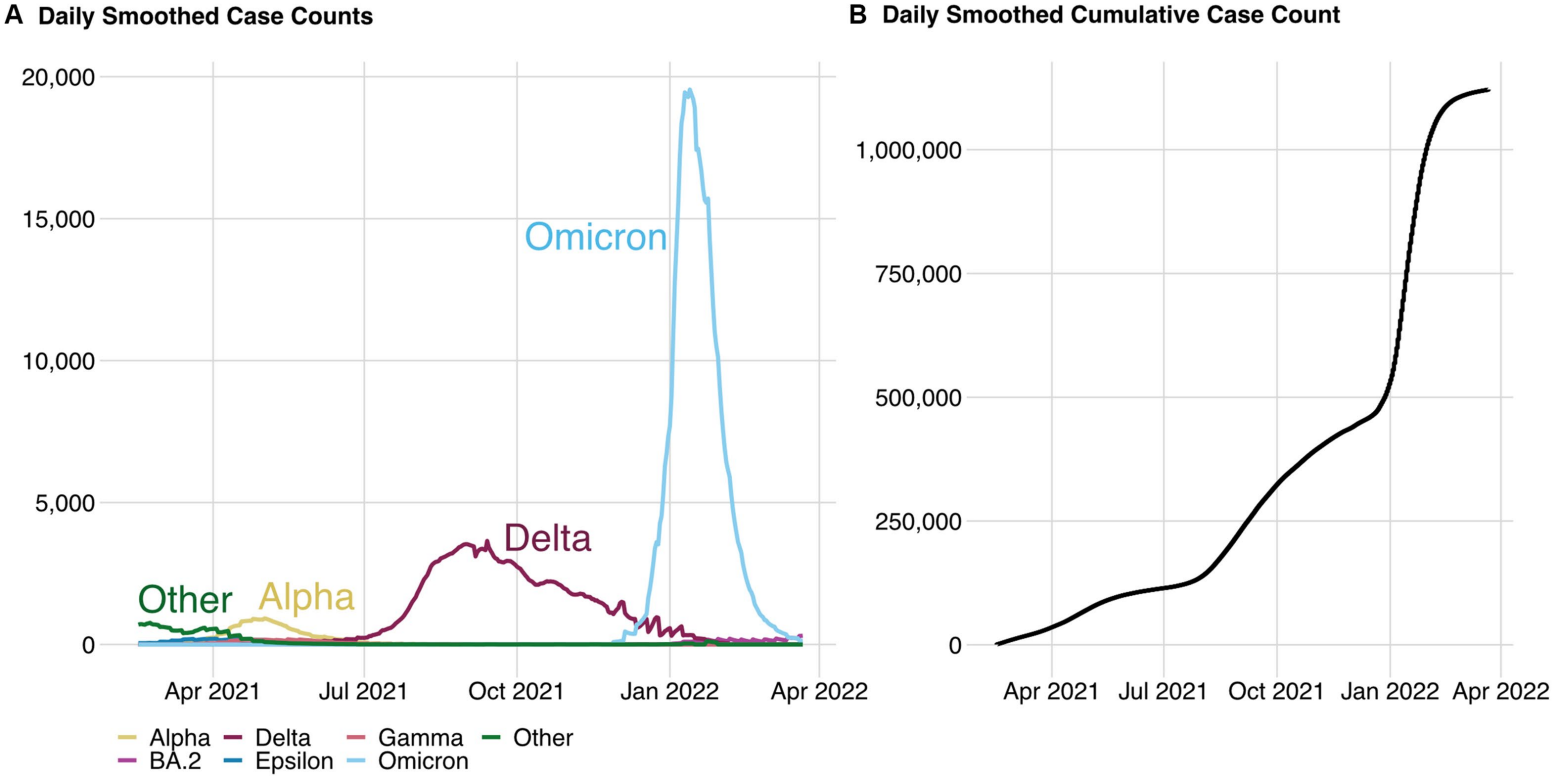
Daily smoothed case counts from probable and confirmed cases **(A)** and the corresponding cumulative daily case counts **(B)** across all variants. Data source: WA DOH.

In Washington State, from 1 March 2021 to 28 February 2022, the WA DOH reported 1,105,000 confirmed and probable COVID-19 cases. Figure 3 shows a smoothed estimate of the daily number of cases for each of seven different COVID-19 variants (on the left) and the cumulative number of cases across all variants (on the right). During the 1-year study period, there were approximately 155,000 cases reported to the ENCV and an estimated 1,089,000 exposure notifications shown on users’ phones.

**Figure 4:**
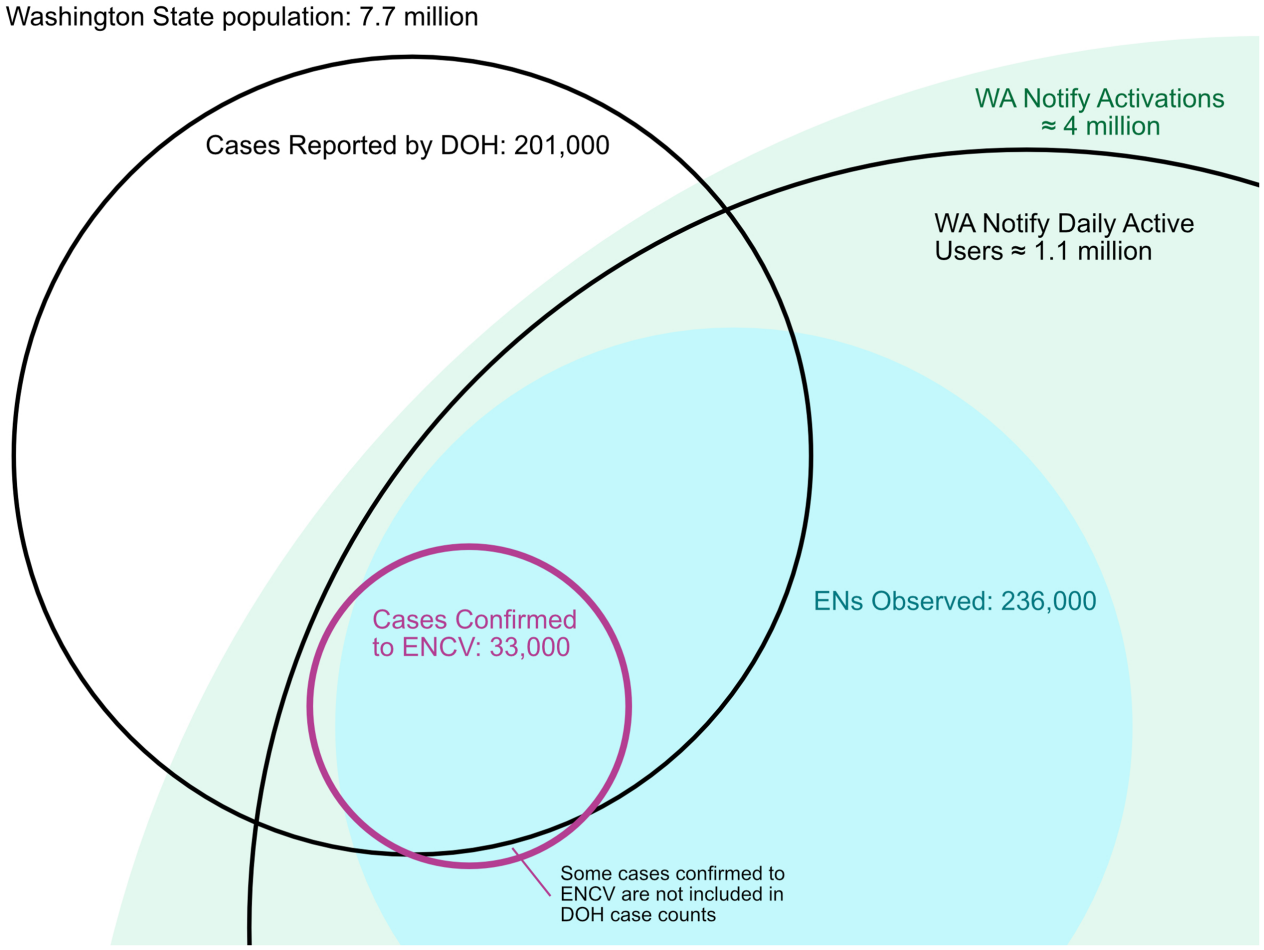
Venn diagram visualizing the number of cases reported by Washington State between 23 January and 19 February 2023, as well as the number of exposure notifications and cases confirmed to WA Notify during the same period. During this period, approximately 8,200 individuals were notified of their positive status by a state case investigator. For context, the estimated number of daily active users during the study period (1.1 million) and total WA Notify activations (approximately 4 million) are shown as well.

For comparison to manual CI/CT efforts reported by the WA DOH (28), the 4-week period from 23 January 2022 to 19 February 2022 is considered and visualized in Figure 4. During this period which includes the peak of the Omicron surge in the state, there were 201,000 cases reported to the WA DOH. Of these cases, 8,200 case investigation interviews were conducted leading to 2,300 contact tracing interviews. During this same period, there were 33,000 confirmed cases registered with the ENCV and a corresponding 236,000 ENs shown on residents’ devices.

**Figure 5:**
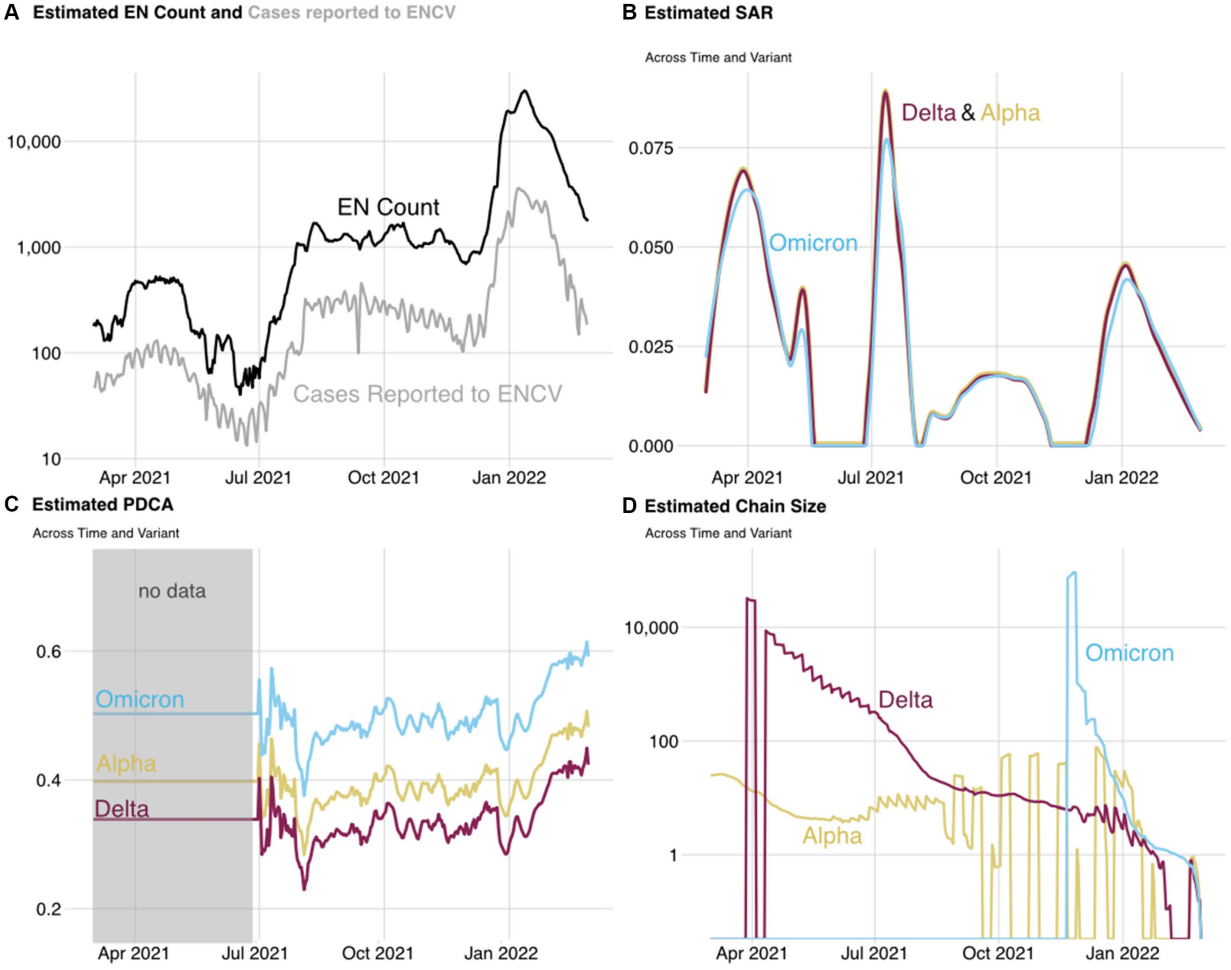
Estimated EN Count **(A)**, SAR **(B)**, PDCA **(C)**, and chain size **(D)** used in the cases averted estimate. Yellow, red, and blue lines represent estimates for the Alpha, Delta, and Omicron variants, respectively. The estimated SAR for the Alpha and Delta variants visually overlaps throughout the study period. Low rates of transmission and added noise to the SAR estimate result in values slightly greater than or less than zero during periods where the true SAR would be zero. These values are rounded to be exactly zero. The PDCA is estimated using an estimate of the time from exposure to exposure notification. Estimates for exposure to exposure notification times are not available before July 2021 and are estimated using the average across all other dates. More details on the estimation of each parameter can be found in the Supplementary material.

Because of the privacy measures of WA Notify, demographic information was only available for the subset of users who responded to the user survey. Survey respondents tended to be from lower-risk populations than the Washington State population (i.e., more likely to report being white, non-Hispanic, and young) (16). Except for the quarantine adherence estimate (which does not vary across time or variant), each of the other four parameters is estimated for each day in the study period and each variant. The daily estimates of each of the four parameters are shown in Figure 5, with yellow, red, and blue lines representing the Alpha, Delta, and Omicron variants, respectively. These estimates along with estimated quarantine adherence are used to estimate the number of cases of COVID-19 averted on each day of the study period for each variant. Table 1 provides average values of each parameter estimate across three different periods (chosen to reflect the time periods in which each variant was dominant).

**Figure 6:**
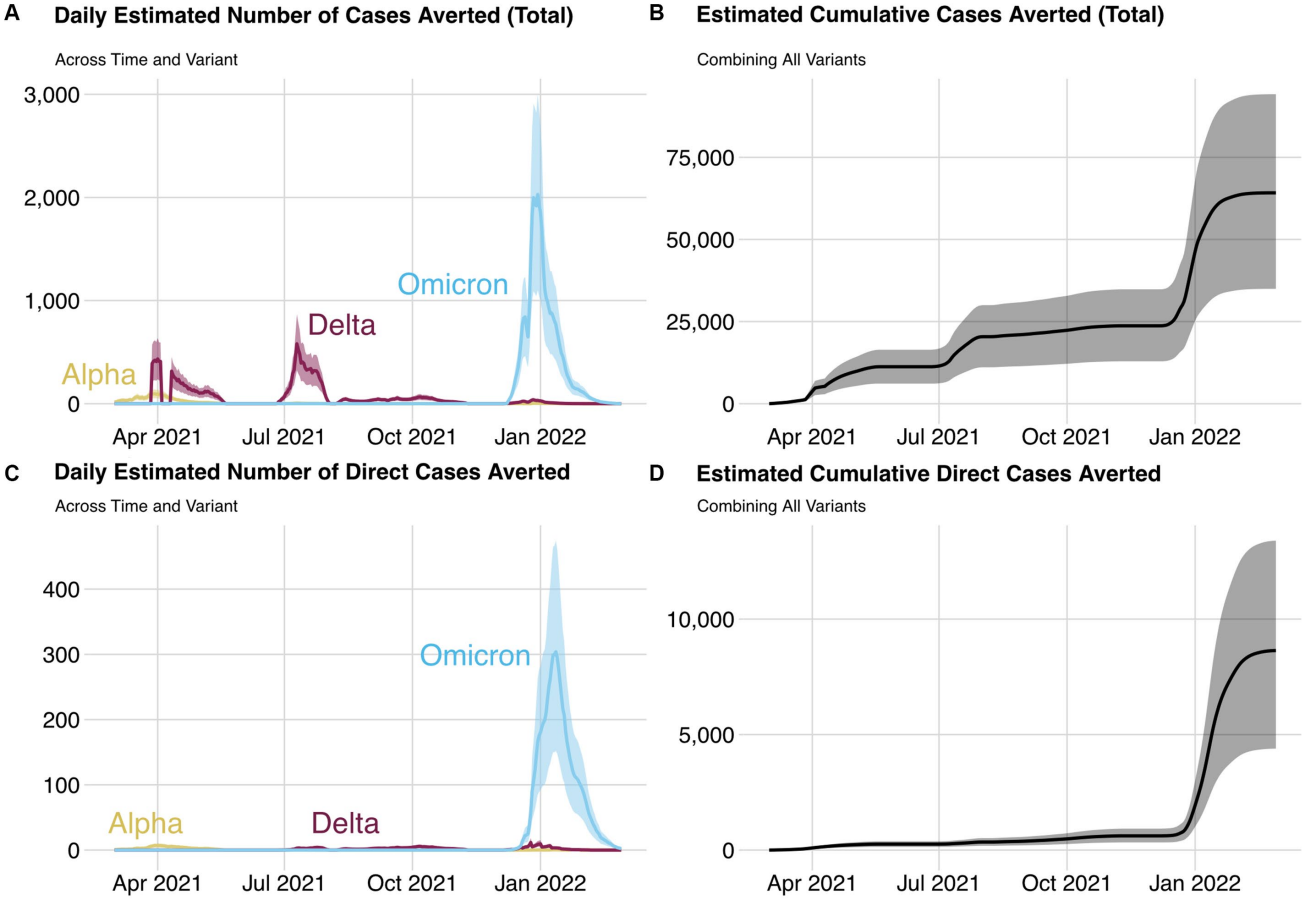
Daily estimated number of cases averted **(A)** and direct cases averted **(C)** by variant and cumulative cases averted **(B)** and cumulative direct cases averted **(D)** for each day and all variants during the study period. The lower and upper bounds of each ribbon are the cases averted estimate from the 30 and 80% quarantine adherence settings, respectively.

The daily, variant-specific estimates of COVID-19 cases averted by WA Notify during the study period, the cumulative count across all variants, and the corresponding counts for direct cases averted are shown in Figure 6. The estimated number of cases averted during the entire 1-year period is 64,000 with an estimate of 35,000 for the 30% adherence setting and 92,000 for the 80% adherence setting. The estimated number of directly averted cases which does not include subsequent infections after the initially averted case is 8,700 (SA 4,400–13,300).

## 4 Discussion

WA Notify saw higher adoption than most digital exposure notification systems in the United States but similar levels to those in Britain. While there were many methods used to evaluate the effectiveness of these systems, our estimate of cases averted is in line with other estimates, given the population size and the level of adoption in Washington State (9, 25, 29). The structure of the model used in this analysis is nearly identical to those of Wymant and Kendall (9, 25) and is similar (but different in some ways) to the analysis of Jeon (29). The Jeon analysis calculates the number of directly averted cases in a manner similar to the one presented here but calculates subsequent cases averted differently. These subsequent cases are calculated by taking the difference between the simulated case counts from a compartmental model under two settings, one with and one without the directly averted cases.

While making definitive one-to-one comparisons between WA Notify and traditional CI/CT methods is not possible, the number of individuals notified by digital exposure notification and CI/CT methods is comparable in scale after accounting for the relative size of the populations served. While the WA DOH released detailed information during the period in which CI/CT capacity was likely most strained, during other periods and in other locations, CI/CT efforts match or surpass those of WA Notify. More detailed comparisons between WA Notify and CI/CT methods are provided in Supplementary Table S4.

The estimator of cases averted used in this analysis was built on a recently developed modeling framework that relies on data from cutting-edge systems created for digital exposure notifications. The structure of these data is different from most public health data sources. Data collected through the ENCV system include all individuals enrolled in the system, providing a census of WA Notify users. Thus, certain metrics (such as code verification counts) are exact. Data collected through the ENPA system include a large, though potentially less representative, subset of the WA Notify users. However, this system provides a rich set of metrics pivotal to estimating cases averted. Metrics from both systems are recorded each day allowing for monitoring of parameters that change over time. Nearly all parameter estimates were partitioned by variant, allowing for modeling of cases averted over long periods in which particular variants appear and disappear (see the Supplementary material for more details).

### 4.1 Limitations

Despite the additional data provided by the WA Notify system, many challenges remain in accurately modeling the number of cases averted by COVID-19 EN systems in the U.S. To maintain privacy, only aggregate counts for each metric, based on users’ interactions with WA Notify, were available. This resulted in at least three limitations of the ENPA and ENCV data that do not exist for survey-based data. First, WA Notify users’ interactions with the ENCV are restricted to a bare minimum, opting-in to the system, reporting a positive test, and publishing keys so the set of available metrics is limited. The lack of demographic information on the study population made evaluating access, equity, and justice issues difficult without additional surveys (30). Second, all metrics in the ENPA dataset include noise that was added to maintain differential privacy. Metrics with small counts (i.e., less than 100) have large variability, and thus, estimators calculated using these metrics have lower accuracy (more details are provided in the Supplementary material). Third, individual-level information is not recorded, and thus, multiple events happening to a single user that occurred at different times were not easily captured (i.e., a single user observing multiple ENs in a single week). While the strong privacy measures advocated for by industry partners were novel to public health and are limiting in some ways, they also likely contributed to the historically high adoption of WA Notify.

To estimate the number of cases averted, there are multiple modeling assumptions that cannot be verified with the available data. Furthermore, it is impossible to empirically determine the direction and magnitude of the bias of the cases averted estimator. More discussion of the assumptions is provided in the Supplementary material.

This article focuses on estimating the number of cases of COVID-19 averted by WA Notify rather than the number of hospitalizations or deaths averted by WA Notify. However, the model outlined here can be extended to estimate these metrics using data available to many state departments of health.

## 5 Conclusion

Smartphone-based EN systems are one of several non-pharmaceutical interventions (NPIs) that were available to public health agencies in the U.S. for COVID-19 pandemic response. But unlike more traditional NPIs, such as case investigation, contact tracing, masking, and social distancing, EN systems are novel, anonymous, public health NPIs. EN systems have sometimes been described as complementary to existing contact tracing/case investigation infrastructure. However, EN systems function differently, having both the disadvantage of lacking human-to-human contact that is sometimes essential to explore individual situations, answer questions, or encourage protective behaviors, and the advantages of anonymous “stranger” notification, greater timeliness, automated notification to avoid contacts who do not answer calls, and a much lower cost to share information about exposure risks.

This study adds to the evidence of the impact of smartphone-based exposure notification systems during the COVID-19 pandemic, measured in cases averted in a single state. Though current NPIs including CI/CT remain valuable tools, we believe that EN systems played a valuable role in the COVID-19 pandemic in high-adoption areas such as Washington State. Further investigations are warranted into the potential of future digital EN systems, including making the system more accessible and equitable, better estimation or (privacy-preserving) measurement of model parameters, extensions to the functionality and public health utility of digital EN systems, and expansion of these systems to other public health problems such as foodborne illness and seasonal flu.

## Data availability statement

The data analyzed in this study is subject to the following licenses/restrictions: some data are controlled by the Washington State Department of Health and are not allowed to be released publicly. Requests to access these datasets should be directed to https://doh.wa.gov/data-and-statistical-reports/health-statistics/data-request-faq or the email chs.datarequests@doh.wa.gov.

## Ethics statement

The studies involving humans were approved by University of Washington Institutional Review Board. The studies were conducted in accordance with the local legislation and institutional requirements. Written informed consent for participation was not required from the participants or the participants’ legal guardians/next of kin because this project was a public health quality improvement/surveillance project and therefore was deemed not human subjects research.

## Author contributions

AE: Formal analysis, Methodology, Software, Visualization, Writing – original draft, Writing – review & editing. CA: Formal analysis, Methodology, Writing – original draft, Writing – review & editing, Validation. LM: Conceptualization, Formal analysis, Software, Validation, Visualization, Writing – review & editing. BK: Resources, Writing – review & editing. AH: Writing – review & editing. LW: Writing – review & editing. DL: Methodology, Software, Writing – review & editing. DR: Writing – review & editing. NP: Writing – review & editing. CS: Writing – review & editing. WL: Writing – review & editing, Data curation, Funding acquisition, Project administration. JB: Funding acquisition, Project administration, Resources, Writing – review & editing.
