# Supplementary material for: Evaluation of the effectiveness of Washington State’s digital COVID-19 exposure notification system over one pandemic year
Source: Front Public Health. 2024 Aug 14;12:1408178. doi: 10.3389/fpubh.2024.1408178 (PMC11349652; doi:10.3389/fpubh.2024.1408178)
Supplement: Supplementary file 1 [file Data_Sheet_1.docx]

Supplemental Material

# WA Notify System Information

**Basic** **ENx operation:** Individuals in Washington state accessed WA Notify by activating the service on their Android or iOS phones. Once activated, the phone exchanged cryptographic keys via Bluetooth with other nearby activated phones. These keys were stored locally on users’ phones along with information about the strength and duration of the Bluetooth signal. When a user tested positive for COVID-19, they could confirm their result in WA Notify using a verification code generated by the national Multi-tenant Verification Server. Verification codes could be issued by Washington state Department of Health (WA DOH) or self-requested in WA Notify. After the code was verified, users were able to upload the Bluetooth keys stored on their phone to the National Key Server. Phones with WA Notify enabled periodically downloaded keys from this server. After a device downloaded the list of recently published (uploaded) keys from the server, it checked its history of key exchanges against this list for any matches and calculated a risk score for the last 10 (previously 14) days. If any day’s risk score met the configured threshold and the classification priority would be increased, an exposure notification (EN) was shown by the phone. Text from these ENs for WA Notify read “You may have been exposed to COVID-19, You have been near someone recently who tested positive for COVID-19. What you do next matters and we’re here to help. Tap to learn more.”. Importantly the history of keys exchanged with other phones was only stored locally on users’ phones. Thus, no other phone or server had sufficient information to determine which, if any, phone had exchanged any of the uploaded keys and thus which phones would generate an EN.

Prior to December 2021, codes were automatically issued by WA DOH only to individuals who tested positive for COVID-19 through a lab or provider-based testing service and provided a mobile phone number with their test. Individuals who tested positive using an at-home test, or did not receive a code, could call the state COVID-19 hotline to request a verification code. Beginning in December 2021, WA Notify users could alternatively request a verification code using their phone and self-attesting to testing positive without receiving a WA DOH-issued code. The usefulness of this feature was a result of the increased availability of at-home, over the counter COVID-19 antigen tests.

**WA Notify risk categories:** WA DOH could categorize exposure risk levels based on the strength and duration of key exchanges between devices. When WA Notify launched in November 2020, there was a single notification type requiring an exposure duration of 15 minutes or greater. For further distinction, a second notification type was added in July 2021 for exposures of 30 minutes or greater, resulting in two exposure types (15–30-minute exposures and >30-minute exposures). In December 2021, two additional types were added for shorter exposures (7.5-15 minutes) and exposures to self-attested positive diagnoses (>15 minutes). After the introduction of the option for self-attestation a large fraction of code verifications were self-attested, indicating widespread availability and use of at home testing.

**Opt-in ENPA participation and differential privacy**: The process by which data were sent to and compiled for the Exposure Notification Privacy Analaytics (ENPA) system was different from that of the Exposure Notification Code Verification (ENCV) system.(1) After activating WA Notify, users had the option to agree to send additional EN related information to the ENPA system. Each day, devices that had opted into the program sent a packet of information to the ENPA servers containing metrics on the number and length of interactions with other WA Notify users. This included the number and type of ENs generated on the phone, counts of the number of code verifications (a code was verified once a user entered the provided code into their phone, or using a link), and counts of keys published (which occured once a user agreed to upload their Bluetooth keys to the key server). The ENPA system employed a decentralized cryptographic system, Prio,(2) which was designed such that no single entity in the system (including the WA DOH) could recover individual level information and even aggregate level information was only recoverable by the MITRE corporation (who hosted information for the WA DOH). For additional security, the user’s phone also added a small amount of random noise to the data before it was encrypted and sent to the ENPA system.

Each packet of information was a binary sequence, with each entry in the sequence corresponding to a different measure. For categorical summary measures, each entry in the sequence corresponded to a different level of the categorical variable. For continuous metrics, the metric was coarsened and transformed into a categorical metric by constructing a set of bins, with each possible value of the continuous metric falling within exactly one bin. To introduce differential privacy, each binary entry of the sequence had a small probability of being flipped from 1 to 0 or 0 to 1. All packets of information were then aggregated on the ENPA servers with (debiased) aggregate statistics provided on each metric reported to the ENPA system. Because each packet had noise added to it, these aggregates were not exact, though the level of inaccuracy for each aggregate was reported by the ENPA system. See section 7 for more details on why smaller counts had relatively larger levels of inaccuracy.

We estimate the percentage of WA Notify users who opted-in to the ENPA by dividing the number of ENPA code verifications by the number of ENCV code verifications. The corresponding percentage changed throughout the study period (March 1st, 2021, through February 28th, 2022) but remained close to 50% for the majority of the one-year study period. Beginning in December 2021, the estimated percentage gradually increased, reaching approximately 75% by February 2022 (See Figure S1).

Figure S1: Estimated percentage of WA Notify users who have opted-in to ENPA, as estimated by the ratio of ENPA codes verifications to the number of ENCV codes claimed. This estimated is especially noisy due to the noise added to the ENPA approximation of the number of codes verified by individuals enrolled in ENPA. The original estimate is truncated at 0 and 1 and a LOESS smoother is used as the daily estimate of ENPA enrollment.

# Model Structure

Averted cases on each day for each variant are modeled as the product of five parameters (referred to here as model parameters). All other parameters are referred to simply as parameters and influence the model through their effect on one or more of the five model parameters. The cases averted on a given day is defined as all cases averted as a result of WA Notify users observing ENs that day and choosing to engage in protective behavior because of the EN (this includes direct cases averted on the given day and subsequent cases that would have resulted during the study period had the initial case not been averted). To estimate total cases averted, the number of cases averted for each day and variant is estimated, and then a sum is taken over all variants and all days of the study period. Each model parameter is a function of calendar date (denoted by *t*) and some are also functions of COVID-19 variant (denoted by *j*).

1. The number of ENs generated by WA Notify users’ phones on day $t$, and resulting from each variant $j$, is $C_{EN}\left( t, j \right)$.
2. The ratio of ENs generated on infected WA Notify users’ phones to ENs generated across all WA Notify users’ phones for variant $j$ on day $t$ is the app-based secondary attack rate (SAR) and denoted by $SAR\left( t, j \right)$.
3. The ratio of the number of infected WA Notify users who quarantine after receiving an EN to the number of infected users receiving an EN (quarantine adherence or QA) on day $t$, denoted by $\mathrm{QA}\left( t \right)$.
4. The proportion of direct cases averted (PDCA) is denoted by $P_{DCA}\left( t, j \right)$ and is defined as follows. Consider two scenarios for an adherent WA Notify user who has an EN generated on their phone. In the first scenario, after the EN is generated the user quarantines, and as a result, causes no future infections. In the second scenario, contrary to reality, after the EN is generated, the same “adherent” user behaves as though no EN was generated (and thus does not quarantine). The PDCA is the number of direct cases caused after the EN is generated in the second (counterfactual) scenario divided by the number of direct cases caused during the entirety of the infectious period in the second scenario. This denominator includes direct cases caused both prior to and after the EN is generated. While direct cases occur before an EN is generated in both settings, only when the EN does not change behavior, do direct infections occur after the EN. Thus, the PDCA is the fraction of "possible infections" that the EN prevents by causing changed behavior at the time the EN was received (but not before).
5. Chain size, denoted by $\mathrm{CS}\left( t, j \right)$ is the expected number of future cases of COVID-19 caused by a single infected individual for variant $j$ from the first calendar day the individual becomes infected, $t$, through the end of the study, February 28^th^, 2022.

The product of the five parameter estimates summed over the entire study period and for each variant is the estimate of the number of cases averted by WA Notify:

$$\sum_{t} \sum_{j} C_{EN}(t, j)\times SAR(t, j)\times\mathrm{QA}\left( t \right)\times P_{DCA}\left( t, j \right)\times\mathrm{CS}\left( t, j \right).$$

For each day and variant, the number of direct cases averted is modeled as the product of the number of ENs, SAR, QA, and PDCA for that day and variant. To translate direct cases averted to cases averted, the number of direct cases averted is multiplied by the average size of the infection chain starting on the day of infection and ending on the last day of the study.

## Modeling Assumptions

The assumptions of the model can be placed into three broad categories. The frist set of assumptions are specific to each model parameter and will be described in detail in the next section.

The second set of assumptions are transportability assumptions. These assumptions stipulate that the parameter estimator has the same expectation with respect to both the study (observed) population and the target population that defines the parameter. As an example, if one wanted to estimate the average height of twelve-year-old’s in Washington state, but only used a sample from a single middle school, then the estimator would implicitly assume that the middle school population was transportable to the rest of the state with respect to height. These assumptions are described in more detail in the next section.

The last assumption is an assumption of mutual independence between the random variables that are parameterized by the model inputs. As an example, only a fraction of individuals who observe ENs are infectious and only a fraction of infectious individuals quarantine after observing an EN. Because of restrictions in data collected by the EN system, only the marginal probability of an EN being sent to an infected individual (SAR) and the marginal probability of any WA Notify user quarantining (QA) are estimated. The joint probability of both occurring for a single individual (which is needed for an EN to prevent a future infection) is not estimated directly. Instead, independence is assumed between the two random variables and the joint probability is taken to be the product of the estimates of the two marginal probabilities. If the estimated probability of an EN being sent to an infected individual was 50% and the estimated probability of a WA Notify user quarantining was also 50%, the estimated probability of both occurring would be as $0.5 \times0.5 = 0.25$. However, even if both marginal probabilities were 50% the true joint probability could be anywhere between 0% (if each individual who was sent an EN didn’t quarantine) and 50% (if each individual sent an EN also did quarantine). The estimate of cases averted will be biased upward the larger the (positive) correlation is between the random variables and will be biased downward the larger the magnitude of the negative correlation is between these random variables. Unfortunately, it is only possible to speculate on the size and direction of these correlations since only aggregate counts are accessible (eliminating the possibility of directly estimating joint probabilities).

# Estimation of the Five Model Parameters

In this section, we review the methods used to estimate each model parameter in the cases averted model. Some of these model parameters are determined using other, secondary parameters (such as the incubation period distribution or the distribution of time from exposure to EN). Descriptions of how these secondary parameters are estimated are provided after this section. Figure S2 displays a summary of the process by which four of the five parameters in the cases averted model are estimated using the available data sources. The remaining parameter (quarantine adherence) is directly estimated using data from the WA Notify User Survey.


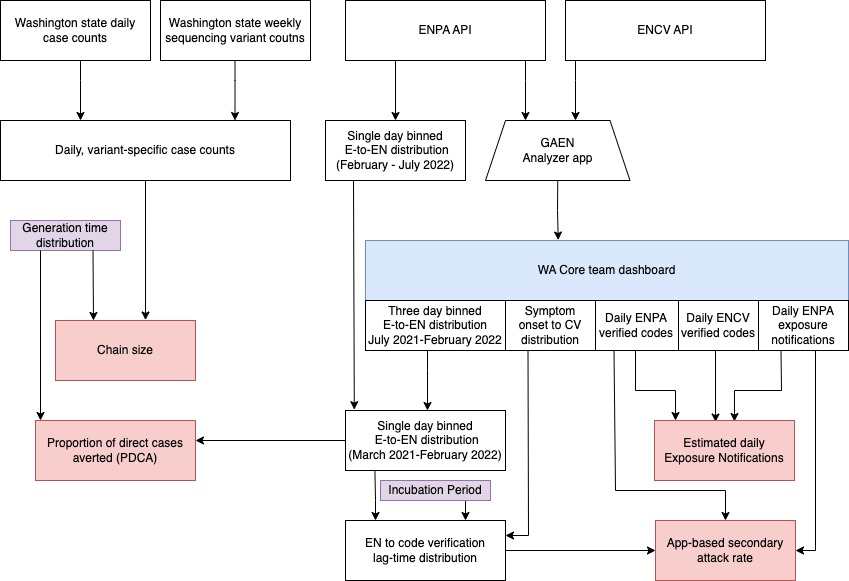


Figure S2: A flow diagram showing the process by which four of the five parameters used in the cases averted model are generated. ENPA stands for Exposure Notification Privacy Analytics and ENCV stands for Exposure Notification Code Verification.

## Daily Number of ENs Generated

The data required to determine if an EN should be generated on a phone were only available on the phone itself, and as a result the number of ENs across all WA Notify users was not directly known. Fortunately, the approximate number of ENs generated on phones for users who had opted-in to the ENPA system is known. To estimate the number of ENs generated across all WA Notify users, the number of ENs generated among ENPA opt-ins is scaled to all WA Notify users. To scale up, it is assumed that the population of WA Notify users and the sub-population of those who have additionally opted-in to the ENPA have the same ratio of codes verified to EN’s generated. That is:

$$\frac{Codes verified by ENPA enrollees}{ENs generated by ENPA enrollee phones}=\frac{Codes verified by ENCV}{ENs generated by WA Notify user phones}.$$

If this assumption holds, we can obtain an unbiased estimate of the number of ENs generated across all WA Notify users’ phones. If this assumption does not hold (which is likely) then the estimate of daily EN counts may be biased. However, determining the direction of this bias is not easy; it is unknown whether the ratio on the left or right in the above equation would be expected to be larger. If those enrolled in ENPA took fewer risks, then it is possible that the relative number of ENs to CVs would be higher for ENPA enrollees since these ENs are generated as a result of low-risk interactions (such as socializing outside). This would suggest that the fraction on the left is smaller. Conversely it could be the case that ENPA enrollees are more likely to get tested and report their diagnosis in the app when infected, which would in turn suggest that the fraction on the left is larger. The estimate for the daily ENs generated among WA notify users utilizes both ENPA and ENCV data and the assumption discussed above.

**Estimating daily ENs generated by ENPA opt-ins:** Data from ENPA is additionally debiased, normalized, and reported as a 7-day rolling average by the GAEN Analytics app.(3) This app provides estimates of the number of ENs generated per day per 100,000 reporting devices and the number of devices reporting EN counts to ENPA. These two values allow for estimation of the number of ENs generated on phones belonging to users who have opted-in to ENPA.

$$Estimated ENs generated by ENPA users' phones=$$

$$\frac{ENs per 100,000 Devices reporting \times Number of devices reporting ENs}{100,000}$$

To estimate the number of ENs generated by all WA Notify users, the number of ENs generated by ENPA users is divided by the fraction of WA Notify users who have opted-in to ENPA.

$$Estimated ENs for WA Notify users = \frac{Approximate ENs reported by ENPA users}{Proportion of WA Notify users enrolled in ENPA}$$

(1)

**Proportion of WA Notify users enrolled in ENPA:** The proportion above is estimated using the ratio of the number of codes verified as reported by ENPA users to all codes verified on the ENCV server (which records all WA Notify code verifications).

$$Proportion of WA Notify users enrolled in ENPA = \frac{Codes Verified by ENPA users}{Codes Verified in ENCV}$$

The number of codes verified by ENPA users is calculated using the product of the number of code verifications received per 100,000 devices reporting code verification counts and the number of devices reporting code verification counts to ENPA, divided by 100,000. The denominator is the product of the number of codes issued by the ENCV system and the proportion of these codes that are verified by users (also called claiming a code). We also assume that each day the proportion of users infected by each variant who have an EN generated on their phone is equal to the proportion of all individuals infected by each variant. For example, if 50% of all cases on a given day are a result of the Omicron variant of COVID-19, we assume that among the cases that receive an EN that same day, 50% are a result of the Omicron variant of COVID-19.

Once both the number of ENs for ENPA users and the proportion of WA Notify users enrolled in the ENPA are estimated the estimate of ENs among all WA Notify users is the ratio of these two estimates, as shown in equation (1).

During the one-year study period, an estimated 1,089,000 ENs were generated, with the estimated daily ENs reaching its maximum on January 12^th^, 2022, with an estimated 30,200 ENs generated that day.

## App-Based Secondary Attack Rate

The app-based SAR on day *t* is the number of ENs generated by WA Notify user phones on day *t* who have been infected with COVID-19 divided by the total number of ENs generated on day *t*. The numerator is estimated using the daily counts of the number of phones that have verified a code (CVs) that have also generated an EN in the past 14 days (referred to here as CVs with an EN). Because only the ENPA system records information on the number of CVs with an EN, data from the ENPA system are used to estimate both the numerator and denominator of the app-based SAR. This estimate is used for all WA Notify users, not just ENPA opt-ins.

Because of the time that elapses between EN generation and code verification (the latter of which can only happen after testing positive), the number of CVs with an EN on a day t (which includes individuals infected on previous days) does not reflect the number ENs generated on phones of individuals who are infected that same day. Taking as a starting point the number of CVs with an EN on day $t$, this lag time is accounted for when estimating the number of ENs generated on infected individuals’ phones who are infected on day $t$. Because the incubation period is different for each variant, it is also expected that the lag time between EN and CV will be different across variant. As a result, the estimated SAR varies slightly between some variants. It is expected that the estimate of SAR will be biased downward since not all those with a generated EN who are infected will go on to test positive and verify a code.

**Estimating CVs that follow an EN**: The count of CVs following an EN is closely related to the number of ENs generated on infected users’ phones. The number of CVs with an EN on each day for each notification type is estimated by the sum of the number of CVs with each of the four notification types, and each of these numbers is calculated as the number of CVs of each type per 100,000 individuals in ENPA multiplied by the number of individuals reporting codes verified data to ENPA divided by 100,000. Similar to the CV count estimates, the estimated number of ENs among ENPA individuals is the product of the number of ENs generated per 100,000 devices reporting EN counts to ENPA and the number of devices reporting EN counts to the ENPA system, divided by 100,000. Both numbers are taken from the ENPA dashboard. After adjusting for the lag time, the daily CV counts provide an estimate of the numerator of the SAR and the daily EN counts provide an estimate of the denominator of the SAR.

**Accounting for lag time:** One difficulty in calculating the app-based SAR is that for a given day *t*, many of the infected individuals who had ENs generated on their phone on day *t* will not test positive and verify a code until some later day. Such verified codes will appear in the counts of a later day, rather than the day *t* on which the EN was generated. Thus, dividing the number of CVs with an EN on day *t* by the number of ENs on day *t* will result in an unsound estimate of SAR.

To account for the time lag between EN and CV, suppose for now that the distribution of time from EN to code verification is known and does not change from day to day. Letting the (unknown) number of ENs generated on infected users’ phones on day $t$ be denoted by $EN_{I}\left( t \right)$, and letting $p\left( t \right)$ be the probability that time from EN to CV is $t$ days, it follows that the expected number of CVs with an EN on day $t$ (denoted by $CV\left( t \right)$) is equal to

$$\sum_{i=0}^{14} {EN}_{I}\left( t-i \right)p\left( i \right).$$

(2)

The above formula will hold for each $t$ so it is possible to solve for $EN_{I}\left( t \right)$ for each $t$ by solving the system of linear equations described above.

Unfortunately, in practice both the number of ENs and the time from EN to code verification are measured with error. Additionally, the system of linear equations has nearly as many (known) equations as unknown quantities. As a result, estimation of $EN_{I}\left( t \right)$ using linear regression results in inaccurate estimates. A simple alternative method for estimating $EN_{I}\left( t \right)$ is to shift back that date of each code verification by the average lag time. In settings where the number of code verifications each day remains roughly constant, this approach will provide a good approximation. Additionally, while this method does not exactly recover $EN_{I}\left( t \right)$ in most settings, it is possible to check how well the estimate aligns with the observed number of CVs with an EN. Figure S3 shows both the observed number of CVs with an EN each day and the number expected given the estimated number of ENs generated on infected users’ phones each day. The number of expected ENs generated on infected users’ phones is calculated using the number of CVs with an EN, an estimate of the lag time distribution and equation (2).


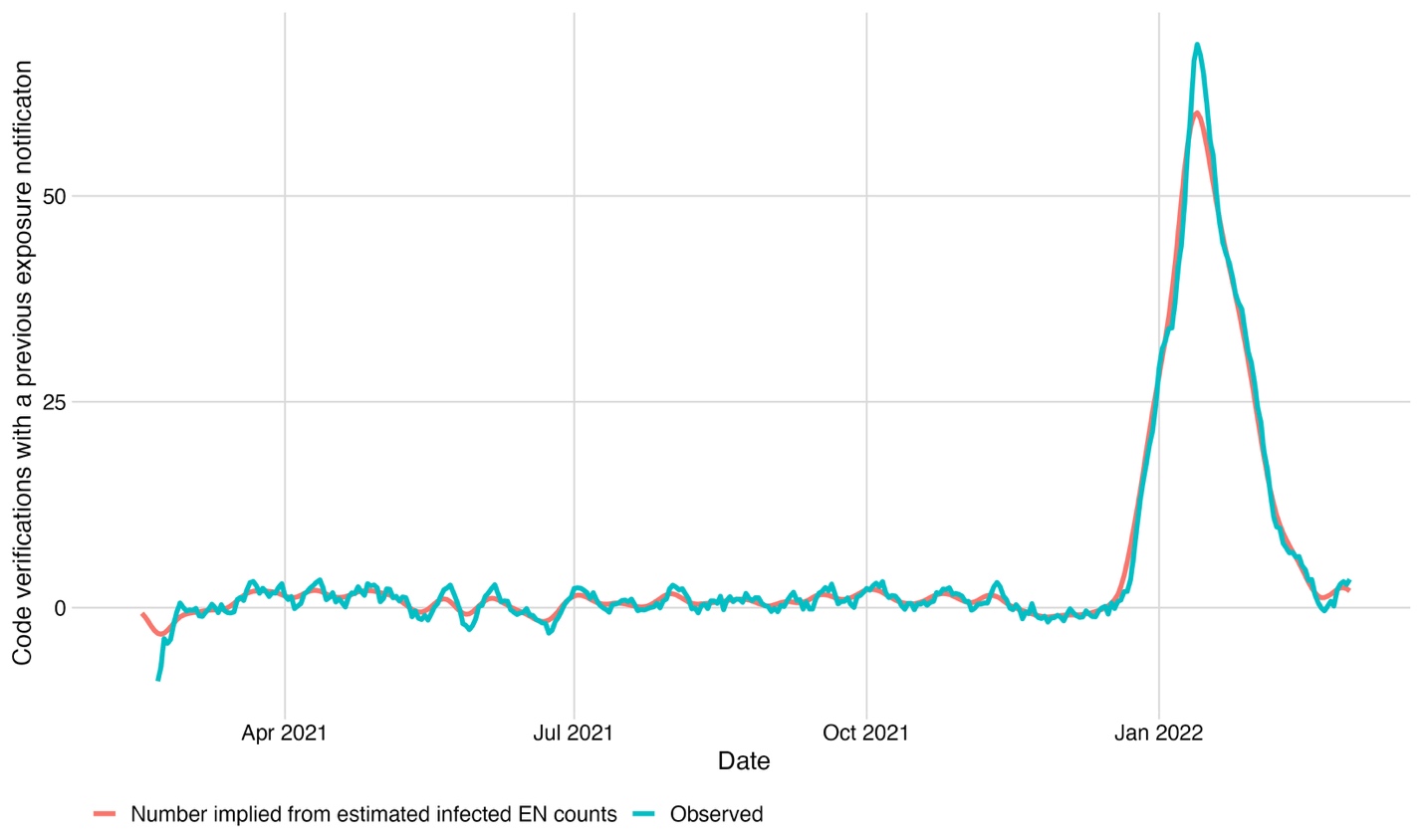


Figure S3: The number of CVs sent to individuals who also have generated an EN in the past 14 days. The blue line shows the observed counts, and the red line shows the number of ENs generated by users’ phones that would be implied given the estimated number of ENs generated by infected users’ phones each day.

While there is a different estimated SAR for each variant, a simplified set of variants is considered when summarizing SAR and all model parameters. The Delta variant is considered by itself, all subvariants of Omicron are considered together and all other variants sequenced by the WA DOH are grouped together as the “Alpha” variant. While there is overlap in the periods in which each variant was present, we classify the period from March 1^st^ to June 31^st^, 2021, as the Alpha period, the period from July 1^st^, 2021, to November 30^th^, 2021 as the Delta period, and the period from December 1^st^, 2021 to February 28^th^, 2022 as the Omicron period. During these variant periods, the average estimated SAR was 3.7%, 1.6%, and 2.2% for the Alpha, Delta, and Omicron variants, respectively. This and other estimated parameters are shown in Figure 4 and Table 1 of the main text.

## Quarantine Adherence

The model accounts for the fact that only a subset of the infected individuals with a generated EN on their phone will take protective measures during the infectious period after the notification. Even early in the pandemic with stay-at-home measures in place, many individuals were unable to, or chose not to, quarantine after an EN was generated on their phone. Quarantine adherence (QA) is important in modeling the effectiveness of WA Notify but is difficult to estimate, especially when considering its time-varying nature.

Quarantine adherence is estimated using questions regarding protective behaviors that were asked as part of the WA Notify user survey. This estimate (based on survey responses) could be biased upwards because of both sampling bias and social desirability bias. Conversely, the estimate of QA may be biased downwards due to differences between the population of interest and the surveyed population. The population of interest includes only individuals with an EN generated on their phone who are also infected whereas the surveyed population includes all participants with a generated EN. The population of interest may be more likely to take protective behaviors since they are more likely to have symptoms and have been in high-risk settings than the study population. QA has likely changed as vaccines became available and the perceived and actual morbidity and mortality associated with COVID-19 changed over the course of the pandemic. Additionally, guidance regarding protective behaviors following an exposure have changed throughout the pandemic (e.g., whether quarantine is recommended, length of recommended quarantine time). Therefore, multiple levels of QA are considered in the model, though our primary estimate of cases averted uses a QA of 55.8% based on the estimate of protective behaviors calculated using survey data.(4) While the estimated QA changes throughout the study period, the size of the fluctuations is small enough that QA is assumed to be constant when estimating the number of cases averted.

## Proportion of Direct Cases Averted

While ENs may offer an early warning sign of infection, it is possible that the EN will be generated at some point after the exposed user has become infectious. If this is the case, then even if the notified individual isolates for the remainder of the infectious period, it is possible they could have caused infections prior to the EN being generated. This possibility is accounted for in the model with the proportion of direct cases averted (PDCA) term.

For an EN to prevent a subsequent infection, the time between original infection and potential subsequent infection (also called generation time) must be longer than the time between original infection and EN generation. For individuals who adhere to quarantine, if the generation time is longer than the exposure to EN time then the model counts the potential infection or new case as averted. The PDCA among WA Notify users who quarantine is modeled as the probability that the time from infection to EN is less than the generation time. This assumes that individuals who adhere to quarantine will do so immediately after the EN is generated. This assumption introduces an upward bias of our cases averted estimate when it does not hold. The two distributions required to estimate the probability that time from exposure to EN is less than generation time are estimated as follows:

- the estimated distribution of generation time for each variant is taken from prior studies and is assumed to be fixed over the study period,(5,6) and
- the distribution of time from exposure (infection time for those that are infected) to EN is estimated for each calendar day using data from the ENPA system and is assumed to not change between the different variants.

**Generation Time:** Following previous work,(7) the distribution of generation time is taken to be a Weibull distribution, with the mean value of the Weibull distribution set to 5.50 days for the Alpha variant, 4.70 days for Delta (5) and 6.84 days for Omicron.(6) A sensitivity analysis is carried out to understand the impact of the assumed generation time on the estimate of cases averted.

**Exposure to EN Time:** Because the distribution of time from exposure to EN is estimated using all individuals (rather than just adherent individuals), it is assumed that non-adherent and adherent users have the same exposure to EN time distribution. The distribution of time from exposure to EN is estimated using data from the ENPA system and more details on how estimation is carried out can be found later in the Supplemental Material.

With estimates of both distributions, the probability of interest may be calculated for each day and each variant. Using these two estimated distributions (and assuming their independence from each other), the estimated PDCA for each variant during their respective time periods are 0.39, 0.32, 0.53 for the Alpha, Delta, and Omicron variants, respectively. The high value of PDCA during the Omicron period can be attributed to the longer estimated generation time of the variant and the shorter estimated time from exposure to EN during that period.

## Transmission Chain Size

The chain size for the period between two time points is the number of new infections that occur between them, originated by a single index case infected at the first time point. When estimating chain size, the start and end time of a chain can significantly impact the size of the chain size. The COVID-19 pandemic has spanned multiple years, and during this time multiple variants of differing transmissibility have circulated throughout the state. To calculate chain size, each variant is considered separately, and it is assumed that each chain of infections contains only a single COVID-19 variant. Except for the rare exception when certain mutations occur, each chain of infections contains a single variant. Ignoring this fact would result in the modeled chains spanning multiple variants and would cause a large upward bias of the estimated chain size.
 To estimate chain size, we used a method similar to that used by Ferreti et al.(8) Both our method and the cited method use daily case counts to estimate chain size for a given time span and variant. Like the cited approach, our method assumes that:

- Each other COVID-19 intervention that was in place would have been in place had WA Notify not been used,
- The number of infections prevented by WA Notify is small enough that it would not affect the natural progress of the disease in the population,
- All individual chains of infection prevented by WA Notify are mutually separated.
- The transmission of the virus to persons outside of Washington state can be ignored, as well as the transmission of the virus from persons outside of the state to those using WA Notify.

The population average of the size of the infection-chain starting on day $t_{1}$ and ending on day $t_{2}$ is the number of individuals who have become infected after $t_{1}$ and up and to $t_{2}$ divided by the total number of infected individuals on $t_{1}$. We estimate this average, by counting all the cases for a single modeled chain that occurs during the period of interest ($t_{1}$ to $t_{2}$). Starting at $t_{1}$, we estimate the initial cases of COVID-19 caused by a single individual who was infected at time $t_{1}$. To estimate this value, one could compare the number of infected individuals present at $t_{1}$ to the number of infected individuals $t_{g}$ days later, where $t_{g}$ is the generation time. If, for example, there are twice as many infected individuals on day $t_{1}+t_{g}$ than there were on day $t_{1}$ then intuitively, each infected individual on day $t_{1}$ infected, on average, 2 other individuals. On average, infected individuals present on day $t_{1}$ are no longer infectious on day $t_{1}+t_{g}$. Additionally, on average, each infected individual present on day $t_{1}+t_{g}$ was directly infected by someone who was infected on day $t_{1}$. This suggests that no systematic overcounting or undercounting of chain size occurs. To estimate the ratio of currently infected individuals on day $t_{1}+t_{g}$ to $t_{1}$, the ratio $C\left( t_{1}+t_{g} \right)/C\left( t_{1} \right)$ is used. To motivate use of this fraction, note that number of currently infected individuals on day $t$ will roughly equal $C\left( t \right)\times t_{g}$ since each infected individual from day $t$ would have been infected on one of the $t_{g}$ previous days. Thus, the ratio of currently infected individuals on day $t_{1}+t_{g}$ to day $t_{1}$ can be estimated as the ratio of $C\left( t_{1}+t_{g} \right)\times t_{g}$ to $C\left( t_{1} \right)\times t_{g}$. This fraction will be a good approximation so long as the number of daily cases does not fluctuate too rapidly on the time scale of $t_{g}$. In conclusion, the number of individuals directly infected by a single case infected on day $t_{1}$ can be estimated as $C\left( t_{1}+t_{g} \right)/C\left( t_{1} \right)$.

Similarly, each of the $C\left( t_{1}+t_{g} \right)$/$C\left( t_{1} \right)$ individuals currently infected on day $t_{1}+t_{g}$ will, on average, directly infect $C\left( t_{1}+2t_{g} \right)/C\left( t_{1}+t_{g} \right)$ other individuals. For the period between $t_{1}+t_{g}$ and $t_{1}+2t_{g}$ the index case will have caused (indirectly)

$$\frac{C\left( t_{1}+t_{g} \right)}{C\left( t_{1} \right)}\times\frac{C\left( t_{1}+2t_{g} \right)}{C\left( t_{1}+t_{g} \right)}=\frac{C\left( t_{1}+2t_{g} \right)}{C\left( t_{1} \right)}$$

cases. Adding together the cases from the two time periods ($t_{1}$ to $t_{1} + t_{g}$ and $t_{1} + t_{g}$ to $t_{1}+2\times t_{g})$ the chain size for period between $t_{1}$ and $t_{1}+2t_{g}$ will be roughly equal to $C\left( t_{1}+t_{g} \right)/C\left( t_{1} \right)+C\left( t_{1}+2t_{g} \right)/C\left( t_{1} \right)$. Summing over each of the time periods between $t_{1}$ and $t_{2}=t_{1}+dt_{g}$, the total chain size for the entire period can be estimated using

$$\sum_{i = 1}^{d} \frac{C(t+i\times t_{g})}{C(t)} .$$

(3)

Finally, to calculate the chain sizes for days in-between the discrete timepoints, a linear interpolation is used. We have tested this approach against the approach of Ferretti and found it to provide similar results when the number of cases is stable on the time scale of $t_{g}$. In settings where exponential growth occurs the approach of Ferretti is less accurate than our method. Because the reproduction number for COVID-19 remains relatively close to 1 during the majority of the study period, the differences in the estimates of chain size between our approach and that of Ferretti are not substantial. The work of Pellis L, et al. (9) has shown that discrete methods similar to the ones outlined here can provide a good approximation of the final size of a pandemic even in settings when the pandemic is not actually discrete. Formula (3) is used in the model to estimate the chain size between two days. As discussed earlier, the estimate of chain size assumes that infection chains will not cross one another or themselves. This strong assumption, if broken, could lead to large amounts of bias in our estimate of cases averted. While this assumption is not expected to perfectly hold in practice, the degree to which it is broken has important implications for the accuracy of our estimate. Time periods in which the prevalence of COVID-19 is highest are also the periods in which assumptions around independent chains are likely to be the least accurate.

During the periods in which a variant is first being measured in Washington state, the modeled size of the chain starting that day and ending on the last day of the study period will be very large. This can be seen in denominator of the fraction in the formula for the calculation of chain size shown in equation (3). However, during these early stages, most cases will result from other variants because of the small number of infections of the newly appearing variant. The estimate of case averted accounts for these two factors. The product of the five model parameters includes the chain size (which can be large when a new variant begins circulating) and the proportion of infections that are due to the given variant (which is small when a new variant begins circulating).

# Estimating Secondary Parameters

## Distribution of Time from Exposure to EN

The estimated exposure to EN time distribution (E to EN) is used when estimating two model parameters: the SAR and the PDCA. In the calculation of SAR, the lag time between exposure and CV is needed when estimating the number of ENs generated each day on infected users’ phones. When estimating the PDCA, the E to EN distribution is used to estimate the probability that an EN is generated before an infected individual causes a subsequent infection. The E to EN distribution is estimated for each day of the study period with single day bins. That is, for each calendar day *t* and day count *i*, we calculate p(*t, i*), the proportion of ENs generated on calendar day *t* that are generated *i* days after exposure where $i$ is equal to or in-between 0 and 14.

The data used to estimate this distribution come from the ENPA system and are debiased using MITRE’s debiasing algorithm, with two distinct datasets used to construct the final estimate over the entire year. The first dataset is generated using a custom implementation that provides estimates of the distribution with single day bins, though this estimate is only available for a short period of time near the end of the study period. The second dataset is the product of the GAEN Analytics implementation and has larger bins but is available over a larger time period. In these calculations, each dataset provides information on the number of each of the four notification types: Self-Request (15min+), Advisory (7-14min), Extended (30min+), and Standard (15-29min).

The first data source provides the distribution of time from E to EN using one day binning. These data are available for the dates from February 10^th^, 2022, through July 9^th^, 2022. These data are directly used for the dates from February 10^th^, 2022, through the end of our study period (February 28th, 2022). To estimate the exposure to EN distribution on each calendar day in this period, the proportion of reported notifications (of all types) on that day that were generated *i* days after exposure is the estimate of p(*t, i*).

The second data source provides a coarser version of this information in which instead of single day bins, the bins are 0 to 3 days, 4 to 6 days, 7 to 10 days, and 11 or more days. This coarser data is available from July 1^st^, 2021, through the end of the study period. To generate estimates of the distribution with one day bins, the counts of notifications in each multi-day bin are placed into single day bins based on the single-day binned distribution estimated for the later study period. As an example, if the distribution of E to EN from the first data source had probabilities of E to EN times of 5% for 0 days, 20% for 1 day, 15% for 2 days and 10% for 3 days, then the relative sizes of each bin for the 0 to 3 day period would be 10%, 40%, 30% and 20% respectively. Thus, 10% of the 0 to 3 day ENs would be re-distributed to the 0 day bin, 40% to the 1 day bin, and so on. After this re-distribution is completed (for each type of notification), for each calendar day the proportion of estimated notifications (of all types) that were generated t days after exposure is the estimate of the corresponding probability.

The estimated distribution for the remaining time period (from March 1st, 2021, through June 30^th^, 2021) is the average of the estimated distribution across all dates in which an estimate already exists.

## Lag Time Distribution

To estimate the daily app-based SAR, it is necessary to estimate the distribution of time from EN to code verification referred to here as lag time. While the time from EN to code verification is not directly recorded, it can be estimated using the observed data. For now, assume that for each individual who opts-in to ENPA, and for each infection, they receive an EN before becoming symptomatic and they become symptomatic before verifying a code.

If this is the case, then the time from EN to symptom onset can be estimated by taking the difference between incubation time (infection to symptoms) and infection (exposure) to EN time. Incubation times for each variant are set based on a systematic review and meta-analysis.(10) With the time from EN to symptom onset known, the time from symptom onset to CV would allow for estimation of the desired distribution. Fortunately, an estimate of this distribution is provided by the ENCV system. Thus, the time from EN to code verification can be calculated as it is the sum of time from EN to symptom onset and time from symptom onset to code verification. See Figure S4 for a visual representation of the logic outlined here.


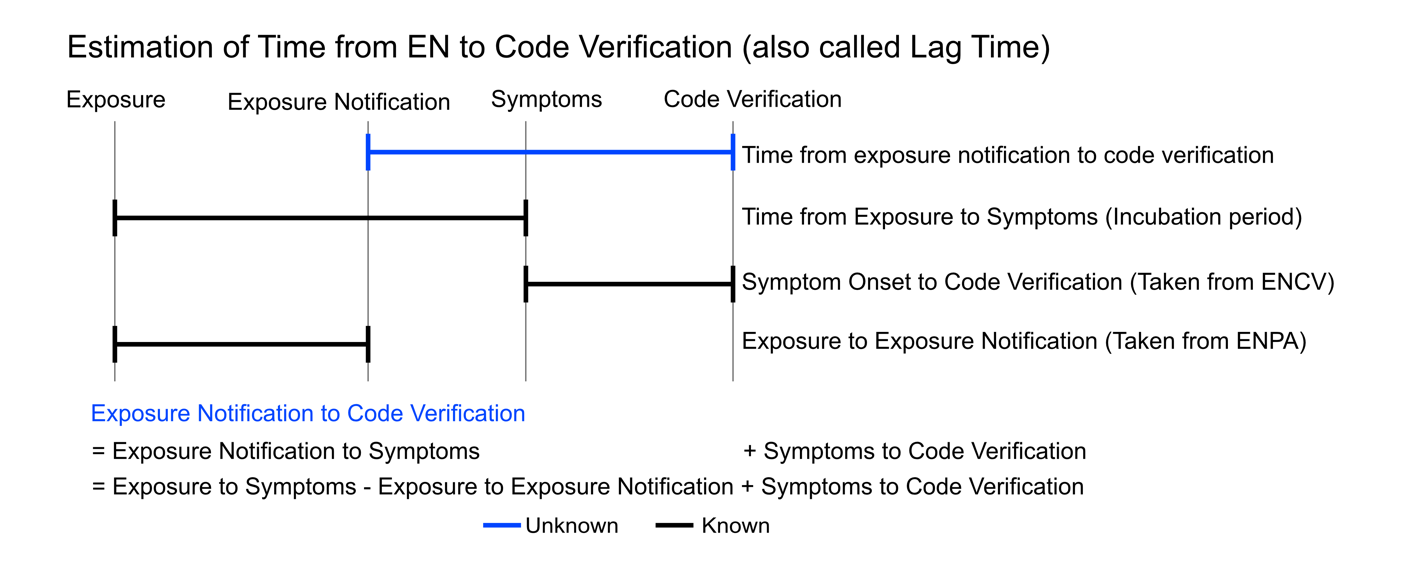


Figure S4: How to estimate lag time from other parameter estimates

## Daily Variant Case Counts and Proportions

The estimate of chain size for each variant on each day is a function of the number of cases of each variant on each day. The number of cases of each variant on each day is estimated using two datasets from WA DOH; the number of sequenced cases of each variant on each day and the total number confirmed and probable cases each day. Sequencing data are available on a weekly basis for each variant provided on the WA DOH website. Because only a subset of all positive cases are sequenced, when estimating the proportion of cases resulting from each variant, counts were averaged across a three-week period to reduce the noise of the estimate. That is, the estimated variant count $\hat{S}\left( t,j \right)$ for week $t$ and variant $j$ is given by

$$\frac{S\left( t-1,j \right)+S\left( t,j \right)+S\left( t+1,j \right)}{3}.$$

Because sequencing data are available both before and after the time period we are studying, this estimate is available for each week considered. The proportion of cases attributed to variant $j$ on week $t$ is given by:

$$p_{var}\left( t,j \right)=\frac{\hat{S}\left( t,j \right)}{\sum_{i=1}^{J} \hat{S}\left( t,i \right)}$$

The rolling average of number of cases $C\left( t \right)$ on day $t$ is provided directly by Washington state, and the estimated number of cases $C\left( t,j \right)$ of variant $j$ on day $t$ is given by

$$C\left( t,j \right)=C\left( t \right)\times p_{var}\left( t,j \right)$$

# Sensitivity Analyses

To better understand how each parameter taken from the outside literature that is used in our model impacts the estimate of cases averted, we conducted multiple sensitivity analyses. The first considers all parameters jointly, the second considers only generation time and the third considers just quarantine adherence.

## Random Draws for Each Parameter

There are three parameters used in modeling cases averted that come from sources outside of WA Notify: generation time, incubation period, and quarantine adherence. While the point estimate for each parameter is used in the main analysis, each was estimated with a reported level of uncertainty in the study in which it was reported. To understand how sensitive our cases averted estimate is to such uncertainty we conduct a sensitivity analysis to understand how the uncertainty of these parameters impact our estimate of cases averted.

We considered (jointly) quarantine adherence, generation time and incubation period. The estimate for cases averted is calculated 10,000 times using values of these parameters that are drawn (independently) at random from a normal distribution. Some parameters are estimated using frequentist methods, and sample estimates are drawn using the (normal) estimated limiting distribution. For the generation time estimates which come from outside sources, the estimate is derived from a posterior distribution which was estimated using Markov chain Monte Carlo methods. Thus, an analytic form for the posterior distribution is not available so draws for these parameters are taken from a normal distribution with a mean equal to the estimate and a standard error equal to the difference between the upper and lower limit of the 95% credible interval divided by 3.92 (2 times the 0.975 quantile of a standard normal distribution). For each replication, each parameter is drawn (independently) from its corresponding distribution and the corresponding number of cases averted is calculated using the randomly sampled parameters. The reported estimate and confidence (or credible) interval for each parameter considered are provided in Table S1.

While quarantine adherence was estimated on a subpopulation of WA Notify users, the survey suffered from sampling bias and the differences between the question responses and quarantine behavior. Neither source of possible bias would be accounted for using a standard confidence interval. We instead used a standard deviation for the distribution of quarantine adherence of 12.5%.

| Variable | Estimate(s) |
| --- | --- |
| Generation Time (5,6) | Alpha: 5.5 (4.7, 6.5)  Delta: 4.7 (4.1, 5.6)  Omicron: 6.84 (5.7, 8.6) |
| Incubation Period (10) | Alpha: 5.00 (4.9, 5.1)  Delta: 4.41 (3.8, 5.1)  Omicron: 3.42 (2.9, 4.0) |
| Quarantine Adherence (4) | 0.56 (0.31, 0.80) |

Table S1: Estimates and confidence intervals for each of the parameters used to calculate model parameters that are taken from an outside data source. Numbers to the right of the variable names are the sources of the estimates and confidence intervals.

Figure S5 is a similar to that of Figure 5 in the main text showing four of the five model parameters (all except quarantine adherence) across each day in the study period. However, the figure also includes bounds for each model parameter (when they exist) that result from the variability in each parameter due to variation in the generation time and incubation period introduced in this sensitivity analysis.


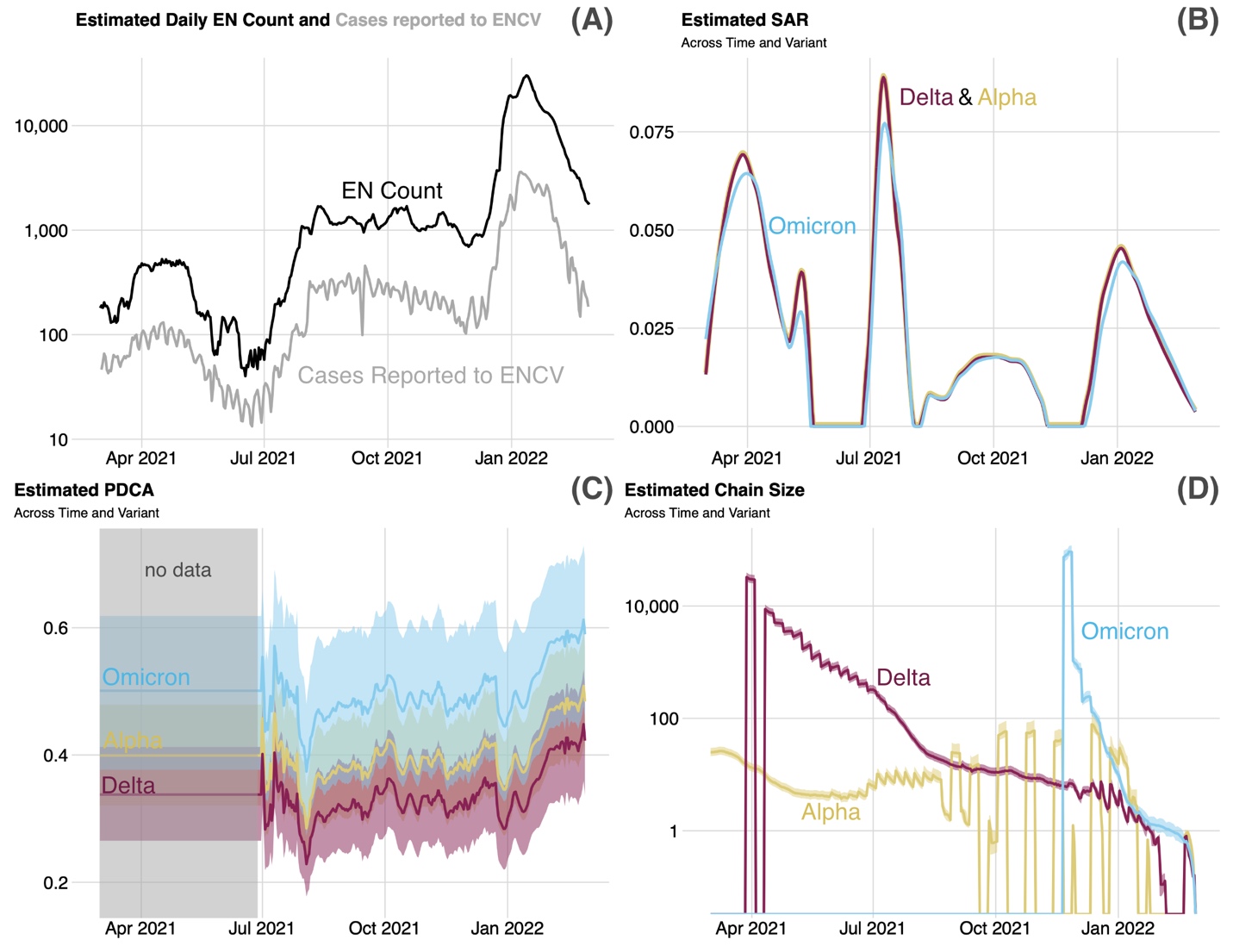


Figure S5: Daily estimates for the estimated daily EN count **(A)**, SAR **(B)**, PDCA **(C)** and chain size **(D)** used in the cases averted estimate. Yellow, red, and blue lines represent estimates for the Alpha, Delta, and Omicron variants, respectively. Ten thousand different models were used with random draws for each of generation time, incubation period and quarantine adherence. The lower and upper bound of each ribbon is the (daily) 2.5% and 97.5% quantile of all estimates, respectively. Each line in each figure corresponds to the median estimate across all ten thousand replicates. The estimated SAR for the Alpha and Delta variants visually overlap throughout the study period. Low rates of transmission and added noise to the SAR estimate result in values slightly greater than or less than zero during periods where the true SAR would be zero. These values are rounded to be exactly zero. The PDCA is estimated using an estimate of the time from exposure to exposure notification. Estimates for exposure to exposure notification times are not available before July 2021 and are estimated using the average across all other dates.

Figure S6 provides a summary of three different sensitivity analyses. The shaded region represents the 95% quantile range of values observed across ten thousand replications for estimated daily cases averted (both direct and overall) described above and the corresponding cumulative cases averted estimates (both direct and overall). The solid center line in each figure panel shows the central estimate based on our estimates for each parameter. The dashed line represents the same quantile range of the same sensitivity analysis except the quarantine adherence level is always set to be equal to 56%. The dotted line shows the primary sensitivity analysis interval from the main text in which adherence levels of 30% and 80% were used (and no other model parameters were varied).


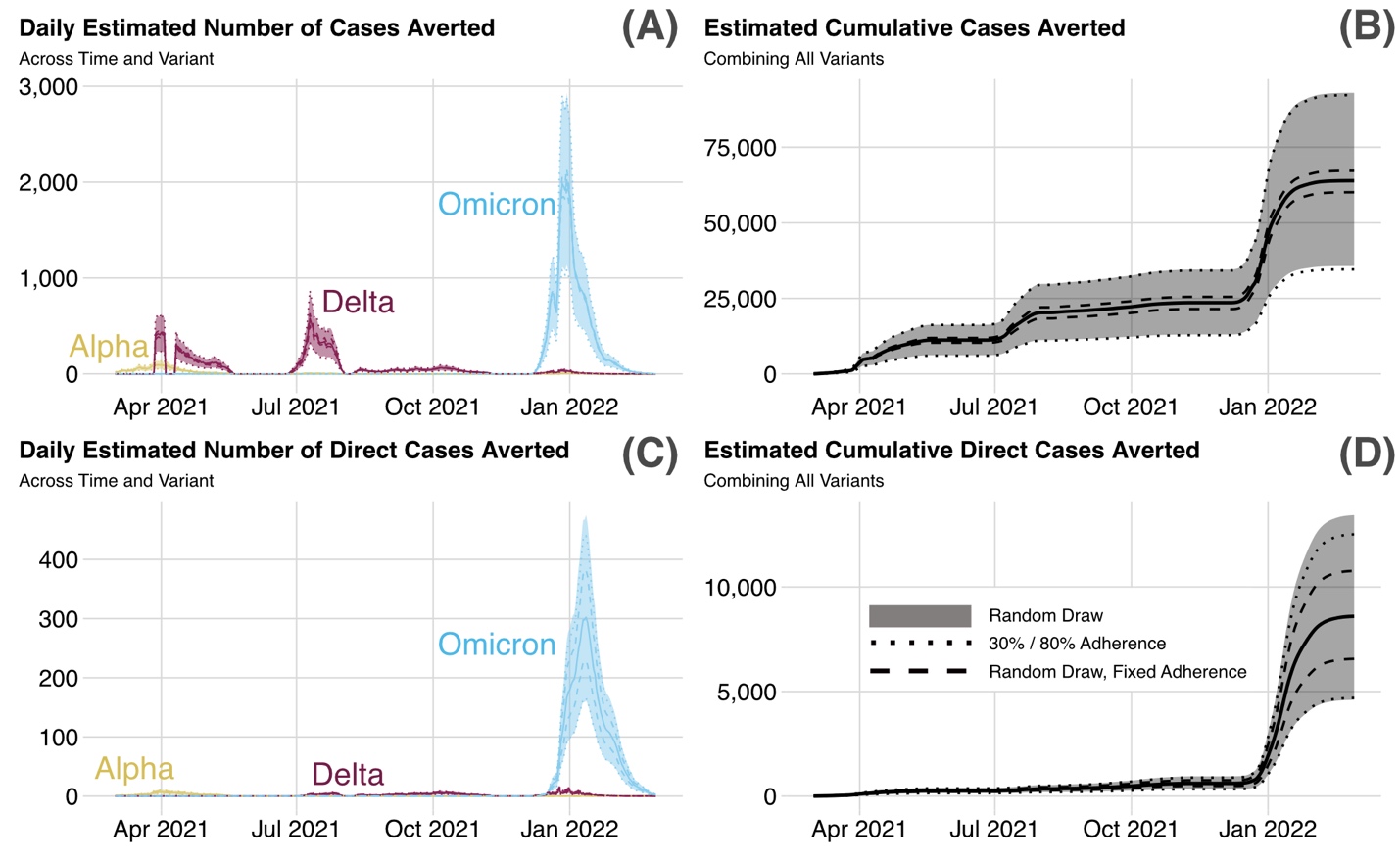


Figure S6: The daily estimated number of cases averted for each variant (for both total (panel A) and direct (panel C) counts) and the estimated cumulative cases averted across all variants (for both total (panel C) and direct (panel D) counts) during the study period. Ten thousand different estimates of cases averted are calculated. For each estimate, each parameter used in the cases averted model is sampled (independently) from the estimated limiting distribution reported in the cited article (see Table S1). The lower and upper bound of each ribbon is the (daily) 2.5% and 97.5% quantile of all estimates, respectively. Dashed lines indecate the same quantile range of a same sensitivity analysis except the quarantine adherence level is always set to be equal to 56%. The dotted line shows the primary sensitivity analysis interval in which adherence levels of 30% and 80% were considered (and no other model parameters varied). Each line in each figure corresponds to the median estimate across all ten thousand replicates.

It is noteworthy how similar the random draw sensitivity analysis is to the primary sensitivity analysis (which only varied quarantine adherence). This suggests that with respect to total cases averted, the uncertainty of model parameters other than quarantine adherence have little relevance. This is also reflected in the small range of values observed in the sensitivity analysis which alters all model parameters except for quarantine adherence.

Surprisingly, this appears to be less true for the direct cases averted analysis, both because there is relatively larger variability in direct cases averted for the sensitivity analysis which keeps quarantine adherence fixed and because the random draw sensitivity analysis appears to be noticeably wider (relatively) than that of the sensitivity analysis that only considers a 30% and 80% quarantine adherence. This is despite the fact that cases averted is a product of direct cases averted and chain size and the relationship between these two variables seems at first to be tenuous. However, as is shown in the next section, changes in generation time have effects on two model parameters with one model parameter increasing and one decreasing. The end result is that these two changes counteract one-another resulting in only small variability in the total cases averted estimate. More details are available in the next section describing why longer generation times result in smaler chain sizes and a larger proportion of direct cases averted. Thus, the sensitivity analyses of direct cases averted (which only are affected by generation time through the higher proportion of direct cases averted) do not have effects that cancel one-another out. This in turn leads in turn to larger variability for estimates of direct cases avert than total cases averted.

## Sensitivity to Generation Time

The generation time of each variant is an important parameter when modeling cases averted, used in the estimation of both the PDCA and chain size. Longer generation times will increase the proportion of infections occurring after EN since a longer generation time results in a longer period when individuals may receive an EN to prevent future infections. Conversely, longer generation times will reduce the estimated chain size. To understand this result, consider a single chain during a fixed time period in which each infection causes exactly one new infection, and the generation time is the same for each infection. This chain of infections takes place over a length of time that is measured as the product of the number of infections and the generation time. If the generation time increases and the time period stays the same, the number of infections in the chain must be smaller. While this example is overly simplified relative to our analysis, similar logic will also apply to the model used to estimate the number of cases averted.

The two effects resulting from a larger generation time (smaller chain size and higher proportion of direct cases averted) oppose each other so it is not clear apriori how modifying the generation time will change the estimate of cases averted. Using the same limiting distributions provided in Table S1, a lower and upper generation time for each variant was considered, where the lower and upper times were taken to be the 10^th^ and 90^th^ percent quantiles of the estimated limiting distributions. These values are displayed in Table S2 and are used for the estimates shown in Figure S7. Using lower estimates of generation time resulted in an estimated 63,000 averted cases and using the upper estimates of generation time resulted in an estimated 65,000 averted cases.

| Variant | Lower | Middle | Upper |
| --- | --- | --- | --- |
| Alpha | 4.9 | 5.5 | 6.1 |
| Delta | 4.2 | 4.7 | 5.2 |
| Omicron | 5.9 | 6.8 | 7.8 |

Table S2: the upper and lower bounds used for the sensitivity analysis of generation time.

Figure S7: Estimated Daily cases averted, and cumulative cases averted across the three sets of generation times listed in Table S2: the upper and lower bounds used for the sensitivity analysis of generation time.

## Sensitivity to Quarantine Adherence

The survey used to estimate the percentage of individuals adhering to quarantine asked respondents about the protective behaviors taken after receiving an exposure notification. Due to changes in local guidance throughout the year of study, the definition of some protective behaviors changed throughout the study period. Table S3 provides a summary of the reportable actions asked about on the protective behaviors question. The action of "stayed home for X days" and "Avoided public places for at least X days" had time periods of 5 days, 10 days or 14 days depending on which survey was being given at the time.

In our main analysis, an individual was considered to be adherent to quarantine if they reported isolating themselves, staying home or avoiding public places for any amount of time, or staying home until they got test results. The survey used to estimate the percentage of individuals adhering to quarantine asked respondents about the protective behaviors taken after receiving an exposure notification. A lower estimate is also included here which does not count individuals as adhering to quarantine if they only reported staying home until getting tested. If the lower estimate was used, the estimated number of cases averted would be 43,000.

| Action | Main Analysis | Lower Estimate |
| --- | --- | --- |
| Stayed home until I got test results | **Included** | Not Included |
| Stayed home for at least X days | **Included** | **Included** |
| Avoided public places for at least X days | **Included** | **Included** |
| Stayed away/isolated myself from others in my household | **Included** | **Included** |
| Contacted my health care provider | Not Included | Not Included |
| Watched for COVID-19 symptoms | Not Included | Not Included |
| I did not do any of the above | Not Included | Not Included |
| Other | Not Included | Not Included |
| Estimated quarantine adherence | 2,969 / 5,319 ≈ 55.8% | 1,986 / 5,319 ≈ 37.3% |

Table S3: Reportable protective behaviors in the survey from the WA Notify implementation and evaluation project. Each column indicates which protected behaviors were counted as adhering to quarantine in both the main analysis and the lower estimate analysis.

# Comparison with other analysis methods

Many studies have reported on either contact tracing / case investigation (CI/CT) efforts or exposure notification systems used during the pandemic. Here, to provide context to our estimates we present a table comparing both traditional CI/CT and digital exposure notification methods used throughout the pandemic. Some are specific to the United States whereas others consider the United Kingdom, Washington state, or specific jurisdictions in Washington State. While traditional (CI/CT) systems are not directly comparable to digital EN systems, to provide an intuitive understanding of the size of each system we provide summary statistics from multiple studies on both methods from various jurisdictions from the United States and UK. Most numbers were taken directly from articles, but some are calculated using the values from the articles. “Notified individuals per week per 10K pop” seeks to provide an intuitive understanding of the size of the exposure notification method relative to the length of time and size of population considered. The equation for this metic is:

$10,000 \times\frac{Number of notifications reported}{Study length in weeks \times Study population size}$ .

While this table is far from definitive or exhaustive, it shows the results found in the present study are in line with others in the area given the size of Washington State and the level of adoption of digital exposure notifications. One noticeable difference between WA Notify and other systems is the relatively high number of exposure notifications observed per cases reported to the system. This could be a result of the high population density of many WA Notify users, or a lower threshold for an EN being shown to the user or a combination of the two.

|  | Traditional CI / CT Systems | | | |  | Digital Exposure Notification Systems | | |  |
| --- | --- | --- | --- | --- | --- | --- | --- | --- | --- |
| Source | Miller et. al, 2021 (11) | Bonacci et. al, 2021 (12) | | Lash et. al, 2021 (13) |  | **Our Study** | Jeon et. al, 2023 (14) | Kendall et. al, 2023 (15) |  |
| Study Area | Five counties in Central Washington | Washington State | | 14 Health Jurisdictions |  | Washington State | Pennsylvania (excluding Philadelphia County) | United Kingdom and Whales |  |
| Study Period | Jun - Jul 2020 | Aug 2020 | Oct 2020 | Jun - Oct 2020 |  | Mar 2021 – Feb 2022 | Nov 2020–Jan 2021 | Oct 2020 – Sep 2021 |  |
| Study Population | 651,281 | 7,700,000 | 7,700,000 | 20,365,511 |  | 7,700,000 | 11,217,925 | 67,000,000 |  |
|  | - | - | - | - |  | 1,000,000 | 356,835 | 15,000,000 | *Daily Active Users* |
| Individuals contacted | 4,987 | 4,600 | 2,166 | 74,185 |  | 155,000 | 390 | 2,138,000 | *Reported Positive Tests* |
| Individuals interviewed | 3,572 | 3,000 | 1,639 | 4,3931 |  | - | - | - |  |
| Close contacts reported | 2,293 | 2,584 | 2,218 | 74,839 |  | 1,089,000 | 233 | 7,005,000 | *Number of ENs* |
| Close contacts per interview | 0.46 | 0.56 | 1.02 | 1.01 |  | 7.03 | 0.6 | 3.28 | *Exposure Notifications per reported test* |
| Notified individuals per week per 10K pop | 2 | 0.15 | 0.07 | 0.91 |  | 0.39 | 0.0086 | 0.61 |  |
|  |  |  |  |  |  | 64,000 | 2-16 | 1,000,000 | *Estimated Cases Averted* |
|  |  |  |  |  |  | 13 | 3.2 | 22 | *Daily active users (% of population)* |
|  |  |  |  |  |  | 83 | 0.02-0.17 | 149 | *Cases averted per 10K pop* |
|  |  |  |  |  |  | 59 | 7-69 | 143 | *Cases averted per 1K ENs* |

Table S4: Contact tracing and cases averted metrics from this and other studies. Columns on the left side of the table summarize studies of traditional contact tracing efforts and columns on the right side summarize studies estimating cases averted of digital exposure notification systems. Rows with both left and right names correspond to rows with comparable metrics (for example “close contacts reported” and “Number of ENs” are placed on the same row to show they can be compared).

# Additional Mathematical details

For the ENPA reported metrics, the level of inaccuracy was higher for metrics with the lower counts because of the added noise. If each individual’s response is given by $X_{i}$ and the noise added is given by $\epsilon_{i}$ then the number of true yeses is given by $Z:= \sum_{i=1}^{N} X_{i}$ and the number of reported yeses is given by

$$Z^{*}:=\sum_{i=1}^{N} {\left( 1- \epsilon_{i} \right)X}_{i}+{\epsilon_{i}(1- X}_{i} )=\sum_{i=1}^{N} X_{i}-{2\epsilon_{i}X}_{i}+\epsilon_{i},$$

where all $\epsilon_{i}$ are identical, mutually independent random variables with binomial distributions.

Treating the $X_{i}$ as fixed (as opposed to being random variables), note that the variance of $Z^{*}$ is does not change with the number of yeses is given by:

$$Var\left( \sum_{i=1}^{N} X_{i}-{2\epsilon_{i}X}_{i}+\epsilon_{i} \right)=Var\left( \sum_{i=1}^{N} {\epsilon_{i}(1- 2X}_{i}) \right).$$

Since each $\epsilon_{i}$ is independent the variance above is equal to

$$\sum_{i=1}^{N} {{(1- 2X}_{i})}^{2}Var\left( \epsilon_{i} \right)=N\times Var\left( \epsilon_{i} \right).$$

It follows then that if the true number of yeses (Z) is smaller, then the variance of the reported number will be relatively larger.

# References

1. Exposure Notification Privacy-preserving Analytics (ENPA) White Paper. 2021.

2. Corrigan-Gibbs H, Boneh D. Prio: Private, Robust, and Scalable Computation of Aggregate Statistics. In: 14th USENIX Symposium on Networked Systems Design and Implementation (NSDI 17) [Internet]. 2017 [cited 2023 Feb 15]. p. 259–82. Available from: https://www.usenix.org/conference/nsdi17/technical-sessions/presentation/corrigan-gibbs

3. billpugh/GAEN-Analytics: A tool to allow public health authorities that have deployed the ENX version of GAEN to access and analyze their ENPA and ENCV metrics. [Internet]. [cited 2022 Nov 20]. Available from: https://github.com/billpugh/GAEN-Analytics

4. Baseman JG, Karras BT, Revere D. Engagement in Protective Behaviors by Digital Exposure Notification Users During the COVID-19 Pandemic, Washington State, January-June 2021. Public Health Rep [Internet]. 2022 Nov 1 [cited 2022 Dec 11];137(2_suppl). Available from: https://pubmed.ncbi.nlm.nih.gov/35915982/

5. Hart WS, Miller E, Andrews NJ, Waight P, Maini PK, Funk S, et al. Generation time of the alpha and delta SARS-CoV-2 variants: an epidemiological analysis. Lancet Infect Dis [Internet]. 2022 May 1 [cited 2022 Oct 20];22(5):603–10. Available from: https://pubmed.ncbi.nlm.nih.gov/35176230/

6. Manica M, De Bellis A, Guzzetta G, Mancuso P, Vicentini M, Venturelli F, et al. Intrinsic generation time of the SARS-CoV-2 Omicron variant: An observational study of household transmission. The Lancet Regional Health - Europe. 2022 Aug 1;19:100446.

7. Ferretti L, Wymant C, Kendall M, Zhao L, Nurtay A, Abeler-Dörner L, et al. Quantifying SARS-CoV-2 transmission suggests epidemic control with digital contact tracing. Science (1979) [Internet]. 2020 May 8 [cited 2022 Oct 20];368(6491). Available from: https://www.science.org/doi/10.1126/science.abb6936

8. Wymant C, Ferretti L, Tsallis D, Charalambides M, Abeler-Dörner L, Bonsall D, et al. The epidemiological impact of the NHS COVID-19 app. Nature 2021 594:7863 [Internet]. 2021 May 12 [cited 2022 Oct 9];594(7863):408–12. Available from: https://www.nature.com/articles/s41586-021-03606-z

9. Pellis L, Ferguson NM, Fraser C. The relationship between real-time and discrete-generation models of epidemic spread. Math Biosci [Internet]. 2008 Nov [cited 2022 Nov 17];216(1):63–70. Available from: https://pubmed.ncbi.nlm.nih.gov/18789950/

10. Wu Y, Kang L, Guo Z, Liu J, Liu M, Liang W. Incubation Period of COVID-19 Caused by Unique SARS-CoV-2 Strains: A Systematic Review and Meta-analysis. JAMA Netw Open [Internet]. 2022 Aug 1 [cited 2022 Dec 11];5(8):e2228008–e2228008. Available from: https://jamanetwork.com/journals/jamanetworkopen/fullarticle/2795489

11. Miller JS, Bonacci RA, Lash RR, Moonan PK, Houck P, Van Meter JJ, et al. COVID-19 Case Investigation and Contact Tracing in Central Washington State, June–July 2020. J Community Health [Internet]. 2021 Oct;46(5):918–21. Available from: https://link.springer.com/10.1007/s10900-021-00974-5

12. Bonacci RA, Manahan LM, Miller JS, Moonan PK, Lipparelli MB, DiFedele LM, et al. COVID-19 Contact Tracing Outcomes in Washington State, August and October 2020. Front Public Health [Internet]. 2021 Nov;9:782296. Available from: https://www.frontiersin.org/articles/10.3389/fpubh.2021.782296/full

13. Lash RR, Moonan PK, Byers BL, Bonacci RA, Bonner KE, Donahue M, et al. COVID-19 Case Investigation and Contact Tracing in the US, 2020. JAMA Netw Open [Internet]. 2021 Jun;4(6):e2115850. Available from: https://jamanetwork.com/journals/jamanetworkopen/fullarticle/2780568

14. Jeon S, Rainisch G, Lash RR, Moonan PK, Oeltmann JE, Greening B, et al. Estimates of Cases and Hospitalizations Averted by COVID-19 Case Investigation and Contact Tracing in 14 Health Jurisdictions in the United States. Journal of Public Health Management & Practice [Internet]. 2022 Jan;28(1):16–24. Available from: https://journals.lww.com/10.1097/PHH.0000000000001420

15. Kendall M, Tsallis D, Wymant C, Di Francia A, Balogun Y, Didelot X, et al. Epidemiological impacts of the NHS COVID-19 app in England and Wales throughout its first year. Nat Commun. 2023 Feb 22;14(1):858.
