# Supplementary material for: Evaluation of the effectiveness of Washington State’s digital COVID-19 exposure notification system over one pandemic year
Source: Front Public Health. 2024 Aug 14;12:1408178. doi: 10.3389/fpubh.2024.1408178 (PMC11349652; doi:10.3389/fpubh.2024.1408178)
Supplement: Supplementary file 2 [file Data_Sheet_2.pdf]

WA Notify Exposure Notification Survey

We are interested in hearing from people who recently received a message from WA Notify that they might have been exposed to someone who tested positive for COVID-19. This survey should take about 5 minutes to complete.

Did you recently receive a message or alert from WA Notify on your smartphone? The message would tell you that you might have been in contact with someone who tested positive for COVID-19.	◯ Yes<br>◯ No
When did you receive this notice?	______________________
Was this alert for a brief exposure? A brief exposure is one that was less than 15 minutes. The message you received would say you had a brief exposure to someone who tested positive for COVID-19.	◯ Yes<br>◯ No<br>◯ Not sure
Is this the first time you have received an alert from WA Notify?	◯ Yes<br>◯ No<br>◯ Not sure
How many alerts have you received?	◯ 1<br>◯ 2<br>◯ 3<br>◯ 4<br>◯ 5<br>◯ 6 or more
Are you currently experiencing any symptoms?	◯ Yes<br>◯ No
Please check any COVID symptoms you are experiencing:	☐ Fever or chills<br>☐ Cough<br>☐ Shortness of breath or difficulty breathing<br>☐ Fatigue<br>☐ Muscle or body aches<br>☐ Headache<br>☐ New loss of taste or smell<br>☐ Sore throat<br>☐ Congestion or runny nose<br>☐ Nausea or vomiting<br>☐ Diarrhea<br>☐ The symptoms I am experiencing are not listed
We would like to hear about your reactions when you first received an alert from WA Notify. Please check up to 3 terms that best describe your reaction.	☐ Afraid/scared<br>☐ Not concerned<br>☐ Nervous/anxious/worried<br>☐ Curious<br>☐ Annoyed/Irritated<br>☐ Confused<br>☐ Relieved<br>☐ Physical symptoms, such as fever, racing heart, headache<br>☐ Motivated to take action<br>☐ None of the above

After you clicked on the alert, the Department of Health (DOH) page called "What to do next" opened. How useful did you find the information on this page?	◯ Extremely useful<br>◯ Very useful<br>◯ Moderately useful<br>◯ Slightly useful<br>◯ Not at all useful
The DOH "What to do next" page contained information about what to do if you've been exposed to COVID-19. Which of the following do you plan to do? (Select all that apply)	☐ Get tested for COVID-19<br>☐ Get tested for COVID-19 only if I develop symptoms<br>☐ Stay home until I get test results<br>☐ Stay home for at least 5 days<br>☐ Avoid public places for at least 5 days<br>☐ Stay away/isolate myself from others in my household<br>☐ Contact my health care provider<br>☐ Watch for COVID symptoms<br>☐ None of the above
Did you also receive a phone call or text message from public health about being exposed to someone who tested positive for COVID-19?	◯ Yes<br>◯ No<br>◯ Not sure
When did public health contact you?	______________________________
How did you first learn that you may have been exposed to COVID-19?	◯ Public health contact tracer<br>◯ WA Notify<br>◯ Someone who tested positive for COVID-19 told me<br>◯ My workplace notified me<br>◯ My school notified me<br>◯ An organization notified me<br>◯ A business notified me
How confident are you that WA Notify will help slow the spread of COVID-19 in the coming months?	◯ Very confident<br>◯ Confident<br>◯ Slightly Confident<br>◯ Neutral<br>◯ Not confident
Would you recommend that others add WA Notify to their phones?	◯ Yes<br>◯ No<br>◯ Not sure
We would like to learn more about your experiences with COVID-19 testing. Please check all of the following that are true for you.	☐ I have used a home self-test kit<br>☐ I know where to get tested for free<br>☐ I have been tested for COVID-19 before<br>☐ I have experienced problems getting a COVID-19 test when I needed one<br>☐ I have never been tested for COVID-19

Please tell us what problems you have experienced with getting a COVID-19 test in the past.	☐ No COVID-19 test appointments were available<br>☐ The lines at the testing site were too long<br>☐ It was hard to get transportation to or from a testing site<br>☐ I was too busy/I could not set aside time to get tested<br>☐ I was concerned about the cost of COVID-19 testing<br>☐ It would take to long to get my test results<br>☐ The information about when to get tested was confusing<br>☐ I wanted to test at home but could not get a home self-test kit<br>☐ Other
What is your age?	◯ 18-24 years<br>◯ 25-34 years<br>◯ 35-44 years<br>◯ 45-54 years<br>◯ 55-64 years<br>◯ 65-74 years<br>◯ 75 years and older
Do you identify as Hispanic/Latinx?	◯ Yes<br>◯ No
What race do you identify as? (Check all that apply)	☐ White<br>☐ Black/African American<br>☐ Asian<br>☐ American Indian/Alaska Native<br>☐ Native Hawaiian/Pacific Islander<br>☐ Other
What gender do you identify as?	◯ Male<br>◯ Female<br>◯ Nonbinary<br>◯ Prefer not to answer
Do you currently live in Washington State?	◯ Yes<br>◯ No, I live in another state<br>◯ No, I live in another country

What county do you live in?	◯ Asotin<br>◯ Benton<br>◯ Chelan<br>◯ Clallam<br>◯ Clark<br>◯ Columbia<br>◯ Cowlitz<br>◯ Douglas<br>◯ Ferry<br>◯ Franklin<br>◯ Garfield<br>◯ Grant<br>◯ Grays Harbor<br>◯ Island<br>◯ Jefferson<br>◯ King<br>◯ Kitsap<br>◯ Kittitas<br>◯ Klickitat<br>◯ Lewis<br>◯ Lincoln<br>◯ Mason<br>◯ Okanogan<br>◯ Pacific<br>◯ Pend Oreille<br>◯ Pierce<br>◯ San Juan<br>◯ Skagit<br>◯ Skamania<br>◯ Snohomish<br>◯ Spokane<br>◯ Stevens<br>◯ Thurston<br>◯ Wahkiakum<br>◯ Walla Walla<br>◯ Whatcom<br>◯ Whitman<br>◯ Yakima
Have you been vaccinated for COVID-19?	◯ Yes<br>◯ No
How many doses of the COVID-19 vaccine have you received?	◯ 1 dose of a 2-dose vaccine like Pfizer or Moderna<br>◯ 2 doses of a 2-dose vaccine like Pfizer or Moderna<br>◯ 1 dose of a 1-dose vaccine like J&J
Have you received a COVID-19 vaccine booster?	◯ Yes<br>◯ No
Is there anything else you would like to say about receiving a WA Notify alert or message? Please use this space for any additional comments.	______________________________
Thank you for completing the survey. The COVID-19 story changes every day. We would like to hear how the pandemic is affecting you. Please enter your email here if you are willing to receive a future survey or participate in other activities around WA Notify. Your email address will only be used for this purpose and will not be shared with any other individual or agency. We value your participation.	______________________________

Exposure Notification Follow-up Survey

Thanks for agreeing to participate in this survey. The information you provide in this survey is anonymous and will not be shared with any other individual or agency. This survey should take about 10 minutes to complete.

PART 1. We would like to hear about your experiences after receiving a message from WA Notify that you might have been exposed to someone who tested positive for COVID-19. Since receiving an exposure notification on [baseline_arm_1][en_date]:

Did you receive a phone call or text message from your local public health department about being exposed to someone who tested positive for COVID-19?
- ◯ Yes
- ◯ No
- ◯ Not sure

When did you receive this call or text message?

____

In the last 2 weeks, have you experienced any of the following COVID symptoms?
- ☐ Fever or chills
- ☐ Cough
- ☐ Shortness of breath or difficulty breathing
- ☐ Fatigue
- ☐ Muscle or body aches
- ☐ Headache
- ☐ New loss of taste or smell
- ☐ Sore throat
- ☐ Congestion or runny nose
- ☐ Nausea or vomiting
- ☐ Diarrhea
- ☐ The symptoms I experienced are not listed

Since you received the alert, did you get tested for COVID-19?
- ◯ Yes
- ◯ No, I didn't get tested because I knew I was positive
- ◯ No, I didn't get tested

- ◯ I used a home self-test kit
- ◯ I got a PCR test only
- ◯ I got a rapid antigen test only
- ◯ I used a home self-test kit and got a PCR test
- ◯ I got both a rapid antigen and PCR test

We'd like to hear why you did not get tested.
- ☐ No COVID-19 test appointments were available
- ☐ The lines at the testing site were too long
- ☐ It was hard to get transportation to or from a testing site
- ☐ I was too busy/I could not set aside time to get tested
- ☐ I was concerned about the cost of COVID-19 testing
- ☐ It would take to long to get my test results
- ☐ The information about when to get tested was confusing
- ☐ Other

Did you test positive for COVID-19?
- ◯ Yes
- ◯ No

Is this the first time you have tested positive for COVID-19?
- ◯ Yes
- ◯ No

We'd like to hear what else you did after learning you might have been exposed to someone who tested positive for COVID-19. Which of the following did you do? (Select all that apply)

- ☐ Stayed home until I got test results
- ☐ Stayed home for at least 5 days
- ☐ Avoided public places for at least 5 days
- ☐ Stayed away/isolated myself from others in my household
- ☐ Contacted my health care provider
- ☐ Watched for COVID symptoms
- ☐ I did not do anything

PART 2. We'd like more about your activities.

Please fill in the blank with the response that best describes your experience using WA Notify.

	increases	decreases	does not change
Having WA Notify on my phone __________________ my likelihood of doing indoor activities when wearing a mask is required	○	○	○
Having WA Notify on my phone __________________ my likelihood of doing indoor activities when wearing a mask is not required or possible	○	○	○
Having WA Notify on my phone __________________ my likelihood of doing outdoor activities when wearing a mask is required	○	○	○
Having WA Notify on my phone __________________ my likelihood of doing outdoor activities when wearing a mask is not required or possible	○	○	○

PART 3. We'd like to learn a little more about you.

What is your employment status?	◯ Unemployed and currently looking for work<br>◯ Unemployed and not currently looking for work<br>◯ Employed full-time<br>◯ Employed part-time<br>◯ Retired<br>◯ Self-employed<br>◯ Unable to work
Are you currently working from home?	◯ Yes, all of the time<br>◯ Yes, most of the time<br>◯ Yes, some of the time<br>◯ Not at all
Are you a student?	◯ Yes<br>◯ No
Are you attending in-person classes?	◯ Yes<br>◯ No
What is the most common type of transportation you use when you go to work or school?	◯ Walk<br>◯ Bike<br>◯ Public transportation<br>◯ Rideshare or van<br>◯ Private car
Are you a health care worker or non-health care worker?	◯ I am a health care worker<br>◯ I am not a health care worker
What is your highest level of education?	◯ No schooling completed<br>◯ Less than high school<br>◯ High school or equivalent like GED<br>◯ Some college<br>◯ Associates degree<br>◯ Bachelor's degree<br>◯ Master's degree<br>◯ Professional degree<br>◯ Doctorate degree
What is your annual household income?	◯ Less than $20,000<br>◯ $20,000 to $34,999<br>◯ $35,000 to $49,999<br>◯ $50,000 to $74,999<br>◯ $75,000 to $99,999<br>◯ Over $100,000<br>◯ Prefer not to say
Do you plan to travel and spend at least one night away from home in the next 4-6 months?	◯ Yes<br>◯ No<br>◯ Not sure
Where do you plan to travel? (Check all that apply)	☐ Within my state<br>☐ Within the country<br>☐ Internationally<br>☐ Not sure

How do you plan to travel? (Check all that apply)	☐ Private car<br>☐ Bus<br>☐ Train<br>☐ Plane<br>☐ Ferry/cruise ship
Is there anything else you would like to say about receiving a WA Notify alert or message? Please use this space for any additional comments.	______________________________
Thank you for completing the survey. The COVID-19 story changes every day. We would like to hear how the pandemic is affecting you. Please enter your email here if you are willing to receive a future survey or participate in other activities around WA Notify. Your email address will only be used for this purpose and will not be shared with any other individual or agency. We value your participation.	______________________________
